# Middle eastern genetic legacy in the paternal and maternal gene pools of Chuetas

**DOI:** 10.1038/s41598-020-78487-9

**Published:** 2020-12-08

**Authors:** J. F. Ferragut, C. Ramon, J. A. Castro, A. Amorim, L. Alvarez, A. Picornell

**Affiliations:** 1 Institut Universitari d’Investigació en Ciències de la Salut (IUNICS) i Laboratori de Genètica, Departament de Biologia, Universitat de les Illes Balears, Carretera de Valldemossa, km 7.5, 07122 Palma de Mallorca, Illes Balears Spain; 2 i3S-Instituto de Investigação e Inovação em Saúde, Universidade do Porto, Rua Alfredo Allen, 208, 4200-135 Porto, Portugal; 3 IPATIMUP-Instituto de Patologia e Imunologia Molecular da Universidade do Porto, Rua Júlio Amaral de Carvalho, 45, 4200-135 Porto, Portugal; 4 Faculdade de Ciências da Universidade do Porto, Rua do Campo Alegre, s/n, 4169-007 Porto, Portugal; 5 TellmeGen, Calle Arquitecto Mora, 5-4, 46010 Valencia, Spain

**Keywords:** Genetics, Population genetics, Anthropology

## Abstract

Chuetas are a group of descendants of Majorcan Crypto-Jews (Balearic Islands, Spain) who were socially stigmatized and segregated by their Majorcan neighbours until recently; generating a community that, although after the seventeenth century no longer contained Judaic religious elements, maintained strong group cohesion, Jewishness consciousness, and endogamy. Collective memory fixed 15 surnames as a most important defining element of Chueta families. Previous studies demonstrated Chuetas were a differentiated population, with a considerable proportion of their original genetic make-up. Genetic data of Y-chromosome polymorphism and mtDNA control region showed, in Chuetas’ paternal lineages, high prevalence of haplogroups J2-M172 (33%) and J1-M267 (18%). In maternal lineages, the Chuetas hallmark is the presence of a new sub-branching of the rare haplogroup R0a2m as their modal haplogroup (21%). Genetic diversity in both Y-chromosome and mtDNA indicates the Chueta community has managed to avoid the expected heterogeneity decrease in their gene pool after centuries of isolation and inbreeding. Moreover, the composition of their uniparentally transmitted lineages demonstrates a remarkable signature of Middle Eastern ancestry—despite some degree of host admixture—confirming Chuetas have retained over the centuries a considerable degree of ancestral genetic signature along with the cultural memory of their Jewish origin.

## Introduction

Jewish communities in the Balearic Islands date back to the fifth century AD^1^. With the Christian conquest of Majorca in 1229, their physical survival was guaranteed, despite social and religious pressures forcing their conversion to Christianity between 1391 and 1435. Consequently, there were officially no more Jews in Majorca nearly 60 years before the Edict of Expulsion by the Catholic Kings in 1492. Many of these converted Jews were integrated in the general population; however, a few families remained in the ghetto and secretly adhered to Judaism, forming a Crypto-Jewish community which was persecuted by the Inquisition (fifteenth–seventeenth centuries)^2^. The last “*Autos de Fe*” in 1691 put a stop to their hidden Jewish religious practices, and this population of convicts and their descendants came to be known as Chuetas, a word probably derived from the Catalan for Jew^3^, with their social stigma and segregation (imposed by their Majorcan neighbours) continuing until the mid-twentieth century. There was a definitive point of inflection when Majorca opened to tourism, as the arrival of newcomers (Spaniards or foreigners) who had no knowledge of the status of Chuetas led to a decrease in anti-Chueta prejudice. Therefore, Chuetas were an isolated population with very scarce intermarriage with the Majorcan host population until recently^4^. One of the most important defining elements of this group is that they bear one of the 15 surnames of converso lineages (Aguiló, Bonnín, Cortès, Fortesa, Fuster, Martí, Miró, Picó, Pinya, Pomar, Segura, Tarongí, Valentí, Valleriola, and Valls) targeted by the inquisitorial sentences for Crypto-Judaism in the last quarter of the seventeenth century^5^. Some of these surnames are common in other Spanish regions, where they are not related to Judaism. In Majorca, however, they have been fixed in the collective memory by their identification as Chueta families.

The rise of modern Population Genetics in the second half of the twentieth century enabled geneticists to endeavour to define the origins and relatedness of Jewish people^6^. Since then, populations with Jewish origin have been analysed by means of uniparental and recombining markers^7–12^, and more recently also through genome-wide SNP arrays^13–18^. The combined analysis of millions of polymorphic markers along the genome have led to greater precision in the clustering of different Jewish groups and to the ability to estimate the Middle Eastern, European, and African components in each group. These analyses reflect that each of today’s Jewish populations is the result of the blending of Middle Eastern and host populations (European, Asian, or African). Regarding haploid markers, on the one hand, male lineages indicate that most Jewish communities share a common Middle Eastern ancestral origin, and remained relatively isolated from neighbouring non-Jewish communities during the Diaspora. On the other hand, mtDNA studies lead to conclude that there are differences in the demographic history of the widespread communities resulting from the Jewish Diaspora in terms of maternal ancestries, indicating different maternal founder effects.

Converted Jews have also been subject of study, either due to their contribution to the host population genetic pool^19–21^, or owing to their isolation and differentiation from their neighbours, such as the Portuguese Crypto-Jew communities in Belmonte and Bragança^22–24^ or the Chuetas in Majorca, the subject of the present study. Chuetas have been previously studied by means of autosomal and X-chromosome markers^25–29^. The results conducted to date demonstrate that Chuetas are a differentiated population that has kept a considerable proportion of its original genetic make-up, especially clear in some markers where Chuetas show polymorphic frequencies of alleles that are very rare in neighbour populations, but not in Middle Eastern populations^30^. However, a certain degree of admixture from and with the host population has also been detected^26,29^.

In this study, we focus on haploid markers in order to investigate the ancestry and demographic history of the maternal and paternal founding lineages of the Chueta population, and to analyse whether cultural isolation has led to the reduction of genetic diversity in mtDNA and Y-chromosome lineages in this population.

## Materials and methods

### Population sampling

For the study of the Y-chromosome, samples from 146 unrelated males were obtained: 100 from the Chueta population, and 46 from Majorca (Balearic Islands, Spain), included as the host population of Chuetas. The Majorcan samples constitute a subset of a larger sample previously genotyped for 12 Y-STRs^31^. For mtDNA analyses, 183 samples were used: 104 non-maternally related individuals from the Chueta population (some of these samples were used in a preliminary analysis in a conference contribution^32^) and 79 Majorcans. All participants provided appropriate informed consent statements, approved by the "*Direcció General de R* + *D* + *I*" (Government of the Balearic Islands, Spain), and the University of the Balearic Islands (procedure AAEE24/2014), following the procedures approved by the Ethics Committee of the University of Porto (N102/CEUP/2012). Anonymity of the recruited samples was preserved during the study. All methods were carried out in accordance with the guidelines and regulations of the Declaration of Helsinki.

### DNA extraction

DNA was extracted by standard phenol–chloroform method and quantified on a NanoVue Plus spectrophotometer (GE Healthcare Life Sciences, Cambridge, UK).

### Genotyping analyses

#### Y-chromosome

**Figure 1:**
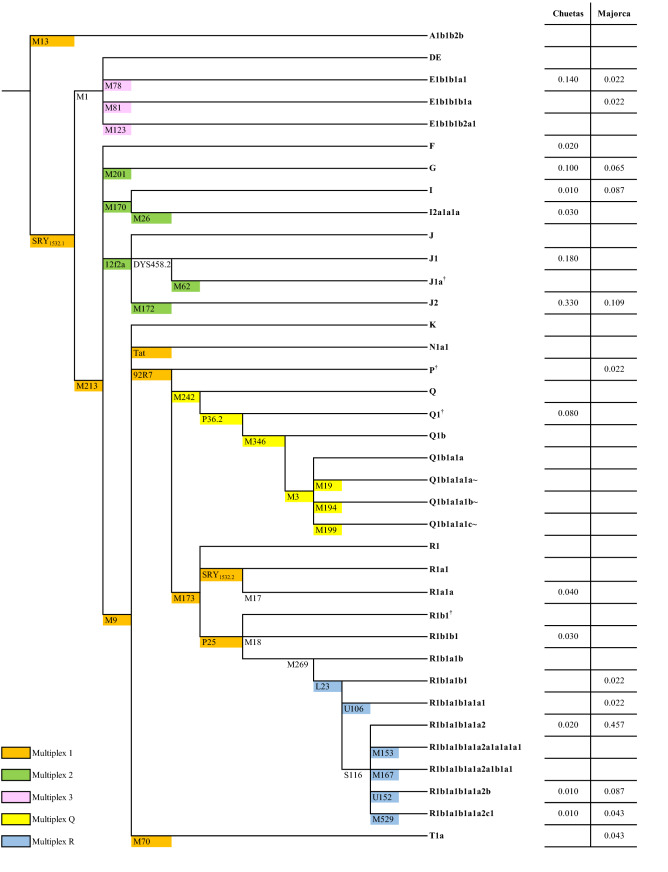
Phylogenetic tree of the 38 Y-SNPs typed, and haplogroup frequencies for Chueta and Majorca populations. Haplogroups were named in accordance with the latest Update of ISOGG 2019. Haplogroups labelled with † are named as in their original description: J1a-M62, P-92R7, and R1b1-P25 (Brion et al.^33^), and Q1-P36.2 (Roewer et al.^35^).

Seventeen Y-chromosome STR markers were amplified using the Y-filer kit (Applied Biosystems, Foster City, CA, USA), following the manufacturer’s recommendations. Thirty-eight SNPs were typed to define the major male lineages. Thirty-two of them were genotyped using SNaPshot kit (Applied Biosystems) in five multiplexes as previously described^21,33–35^ (Fig. 1). M1 and M269 were genotyped with conventional PCR followed by agarose gel electrophoresis; S116, M17, and M18 were genotyped by Sanger sequencing; and DYS458.2 was used to determine the J1 chromosomes^36^.

Y-STR amplification products and Y-SNP purified minisequencing products were separated in an ABI PRISM 3130 Genetic Analyser, and electropherograms were analysed using GeneMapper ID software v3.2 and Peak Scanner software (Applied Biosystems). Y-STR alleles were designated according to ISFG recommendations^37^, and Y-SNP haplogroups according to the latest ISOGG update (2019).

#### Mitochondrial DNA

The mitochondrial DNA control region, or D-loop (16024-576), was amplified with two overlapping fragments using mtDNA-specific primers (L15997, H016 and L16555, H639)^38^. The amplified product was purified with the MBS Spin PCRapace kit (Invitek, Berlin, Germany), and subsequent sequencing reactions were carried out using the BigDye Terminator v3.1 Cycle Sequencing kit (Applied Biosystems), following the manufacturers’ procedures. Finally, products were run in an ABI prism 3130 analyser.

Sequences were assembled and compared to the revised Cambridge Reference Sequence (rCRS) (NC_012920) using Geneious software version 7.1.3 (Biomatters, Ltd., Auckland, New Zealand). Haplogroups were classified following the updated mtDNA phylogeny, PhyloTree, mtDNA tree Build 17 (http://www.phylotree.org/) using HaploGrep2 tool^39,40^, and assigned haplotypes were validated by EMPOP (http://empop.org/) curators.

Moreover, the entire mtDNA molecule was sequenced for six Chueta samples belonging to the modal haplogroup R0a + 60.1 T. Amplification of the whole molecule was carried out by 19 overlapping fragments, and the sequencing strategy used 31 smaller fragments, primers, and specifications as described in Ramos et al.^41,42^. When Chueta specific mutations were identified in the complete mtDNA molecule, the status of such nucleotide positions was interrogated in the rest of the samples belonging to the haplogroup.

### Data analysis

Haplogroup frequencies were estimated by gene counting. Allele and haplotype frequencies, molecular diversity indices, Analysis of Molecular Variance (AMOVA), as well as the corresponding non-differentiation p-values, and Y-chromosome pairwise F_*ST*_ genetic distances were assessed using Arlequin v3.5.1.2^43^. For easier visualisation of the genetic distances observed, a multidimensional scaling (MDS) plot of the pairwise F_ST_ matrix was represented using SPSS v.15.0 (SPSS, Inc., Chicago, IL, USA).

To attain statistical significance for frequencies of putative Jewish founding lineages, Bayesian 0.90 credible region (90% CR) was calculated using SAMPLING software (Macaulay, personal communication). Furthermore, another criterion established by Behar et al.^22^ was to consider haplogroups with a frequency greater than 5% and TMRCA prior to 2 Kya as founder lineages.

Median joining networks of Y-STR haplotypes were constructed using Network 4.6.1.1 (www.fluxus-technology.com)^44^.

In order to visualise the distribution of mtDNA haplogroup R0a frequencies in the Mediterranean geographic context, an isometric spatial frequency distribution map was constructed with the program Surfer 9 (Golden Software, http://www.goldensoftware.com/products/surfer).

## Results and discussion

### Genetic diversity

#### Haplotype diversity

Allele frequencies and gene diversities of each Y-STR of the populations under study are shown in Supplementary Table 1. The DYS385 locus showed the highest gene diversity (GD) values (> 0.83) in both Chuetas and Majorcans, as expected, due to its duplicated structure, while especially low values were found at DYS389I (0.224) and DYS392 (0.288).

Amongst the 146 males analysed, 97 Y-STR haplotypes were observed (Table 1 and Supplementary Table 2), 81 of which were observed only once (singletons). The frequencies of the most common haplotypes were 12% and ~ 4% in Chuetas and Majorcans, respectively. No haplotype was shared between Chuetas and Majorcans. Haplotype diversity in Chuetas (0.965) was considerably lower than in Majorcans (0.998), Bragança Jews (0.987)^23^, or other populations in the literature^45,46^. These results are in accordance with the historical and demographic data of this population and with the reduced genetic diversity in some markers found in previous genetic studies^29,47^.

**Table 1:** Y chromosome molecular diversity indices for haplotypes and haplogroups in Chuetas and Majorcans.

	N	K	UH	HD	h		MPD
**Yfiler**							
Chuetas	100	53	39	0.965 ± 0.008	0.611 ± 0.311	10.387 ± 4.774	
Majorca	46	44	44	0.998 ± 0.005	0.606 ± 0.312	10.308 ± 4.783	
**Y-SNPs**							
Chuetas	100	13	3	0.827 ± 0.023	0.106 ± 0.059	4.033 ± 2.029	
Majorca	47	12	5	0.771 ± 0.058	0.121 ± 0.067	4.614 ± 2.302	

*N* number of individuals, *K* number of haplotypes/haplogroups, *UH* unique haplotypes/haplogroups, *HD* haplotype diversity ± SD, *h* gene diversity over loci, *MPD* mean pairwise differences.

Complete mitochondrial control region haplotypes for Majorcan and Chueta populations are presented in Supplementary Tables 3 and 4. In the 104 samples from the Chueta population, 50 (48.08%) different haplotypes were identified; meanwhile, in the 79 Majorcans, 67 (84.81%) different haplotypes were found. Estimated diversity parameters are summarised in Table 2. Notably, theta k values (θk) in Chuetas were much lower than in Majorcans, but within the same range as those reported for other non-Ashkenazi Jews^22,24^. Therefore, the estimated number of putative female founders in Chuetas is similar to the one estimated in most of these Jewish groups.

**Table 2:** Diversity indices results calculated for the complete D-loop and for the HVRI + HVRII fragment for inter-population comparison.

Population	N	K (% K)	S	HD ± SD	Π ± SD	θk (95% CI)
**D-loop (16,024–576)**						
Chuetas	104	50 (48.08%)	110	0.950 ± 0.015	0.010 ± 0.005	37.216 (25.061; 55.103)
Majorca	79	67 (84.81%)	120	0.995 ± 0.003	0.009 ± 0.005	205.807 (117.911; 374.184)
**HVRI (16,024–16,365) and HVRII (72–300)**						
Chuetas	104	46 (44.23%)	74	0.948 ± 0.014	0.013 ± 0.007	31.003 (20.769; 46.042)
Bragança Jews^24^	57	35 (61.40%)	61	0.967 ± 0.012	0.014 ± 0.008	37.616 (22.328; 63.866)
Belmonte Jews^22^	30	2 (6.67%)	6	0.129 ± 0.115	0.001 ± 0.001	0.279 (0.065; 1.097)
Bulgarian Jews^22^	71	46 (64.79%)	70	0.982 ± 0.007	0.012 ± 0.007	55.477 (34.470; 90.159)
Turkish Jews^22^	123	85 (69.11%)	109	0.985 ± 0.005	0.013 ± 0.007	120.394 (82.638; 176.996)
Libyan Jews^22^	83	36 (43.37%)	63	0.922 ± 0.019	0.013 ± 0.007	23.631 (15.104; 36.717)
Moroccan Jews^22^	148	80 (54.05%)	92	0.979 ± 0.005	0.012 ± 0.006	70.307 (50.484; 97.941)
Tunisian Jews^22^	36	25 (69.44%)	43	0.971 ± 0.015	0.012 ± 0.007	34.939 (18.044; 69.325)
Iranian Jews^22^	82	43 (52.44%)	76	0.971 ± 0.008	0.016 ± 0.008	35.821 (23.098; 55.513)
Iraqi Jews^22^	134	48 (35.82%)	79	0.950 ± 0.009	0.016 ± 0.008	26.344 (18.151; 37.920)

*K* Number of different haplotypes, *S* number of polymorphic sites, *HD* haplotype diversity, *Π* nucleotide diversity averaged over loci, *θk* theta estimator based on the number of different lineages.

#### Haplogroup composition

Regarding the Y-chromosome, Chueta samples were classified into 13 different haplogroups according to the 38 SNPs genotyped (Table 1 and Fig. 1). The six most frequent haplogroups (≥ 4%) (E1b1b1a1-M78 (hereafter E1b-M78), G-M201, J1-DYS458.2 (used in this study as synonymous of J1-M267), J2-M172, Q1-P36.2, and R1a1a-M17) accounted together for up to 87%, with J2-M172 as the most frequent (33%). Three of the seven remaining haplogroups occurred in only one individual. For the Majorca population, 12 haplogroups were found, the most common being R1b-S116 (46%). High diversity values, based on haplogroup frequencies, were found in Chuetas (0.827 ± 0.023) in comparison with other Iberian populations and Majorca (0.771 ± 0.058), but in the same range as the Sephardic Jewish population^19^.

AMOVA analyses at both haplogroup and haplotype (Y-STRs) level showed significance when taking Chuetas and Majorcans as a whole (*F*_ST_ = 0.196; *p* < 10^–5^), indicating the differentiation of the Chuetas with respect to their host population. SAMPLING software identified three differential haplogroups between Chuetas and Majorcans: R1b1a1a2-M269 (hereafter R1b-M269), J1-DYS458.2, and J2-M172, pointing towards the lack of R1b-M269 and the presence of J1-DYS458.2 and J2-M172 as Chuetas' putative founding lineages. The frequency in Chuetas of haplogroups rarely found in neighbouring populations—E1b-M78, Q1-P36.2, G-M201, and R1a1a-M17 (14, 10, 8, and 4%, respectively)—could also mean that they might have been present in the original Jewish Majorcan gene pool.

Network analyses were performed for the main haplogroups (Supplementary Fig. 1). Within networks, Majorcan and Chueta individuals did not share haplotypes. R1b-M269, found mainly in Majorcans, showed the highest diversity (0.998), while the main haplogroups in Chuetas manifested much lower haplotype diversities [ranging between 0.684 (J-12f2a) and 0.833 (R1a1-M17)], except for G-M201 (0.978).

**Figure 2:**
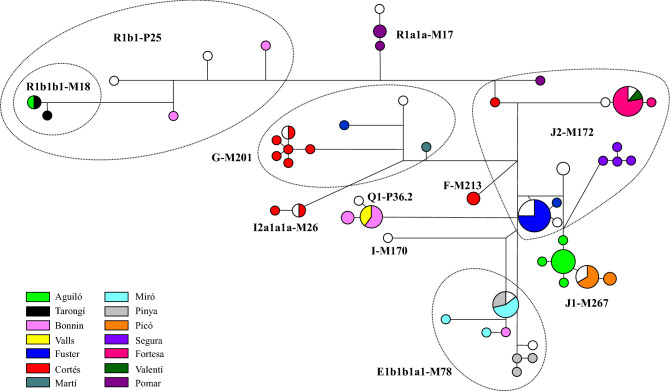
Median-Joining network of Chueta haplotypes. Colour code indicates the Chueta surname of the sample, the colour white is used for anonymised individuals. Smaller circles are singletons and size is proportional to haplotype frequency. Haplogroup assignment is indicated in each group of haplotypes.

Although studying the relationship between haplogroups and surnames was not an initial aim of our work on the Y-chromosome in Chuetas and, therefore, the sampling was not designed for it, with a median joining network we assigned and compared the haplotypes and haplogroups found with the surnames of the individuals (Fig. 2). Fourteen of the 15 Chueta surnames are represented, but not the surname Valleriola, which left no descendants. When sorted and analysed by surnames, the majority of these surname sets contained a highly reduced haplogroup diversity (h = 0.000–0.222), contrarily to that observed in Majorcan non-Chuetas surnames included in other studies^48^. The surnames Bonnín and Pomar revealed two different haplogroups with intermediate h values (~ 0.500) and Cortés was the most diverse with 4 haplogroups and an h value of 0.691; although the sample size would need to be enlarged to confirm these results. Putative founding haplogroups in the Chueta population, J1-M267 and J2-M172, were found to be associated to the surnames Picó and Aguiló (J1-M267) and Segura, Fortesa, and Fuster (J2-M172). The haplotypes carried by the individuals of most surnames show a star-like distribution with only one or two mutational steps between them. Foundation of each Chueta surname by one or a very few individuals in the Christian conversions (fourteenth–fifteenth centuries) could explain these results. In a few cases, the same haplotype is shared by different surnames or, contrarily, individuals within a surname belong to very distant Y-lineages, although the scarcity of historical documents with the Christian names that converted Jews adopted does not allow us to assess the different scenarios that could explain these cases further.

Upon analysing mtDNA results (Table 3), we found the Majorcan population had haplogroup H as the most common one (39%), as was expected of a typical European population^47,49^. Haplogroup H together with haplogroups U, K, and HV (frequencies ranging from 13 to 14%) accounted for 78% of total diversity. Other haplogroups found in this population were I, J, L, N, T, V, and X. In contrast with the Majorcan population, in the Chueta samples, haplogroup H only accounted for 17% of total diversity. The modal haplogroup in Chuetas (~ 20%) was found to be the Middle Eastern haplogroup R0a + 60.1 T, followed by haplogroups T, K, U, and J (19%, 12%, 8%, and 6%, respectively). Together these 5 haplogroups represented 64% of all variation. The remaining lineages were observed at frequencies ranging from ~ 1 to 5% (Table 3).

**Table 3:** Haplogroup frequencies in Chueta and Majorcan populations.

Haplogroup	Chueta	Majorca	Haplogroup	Chueta	Majorca	Haplogroup	Chueta	Majorca
D1j	0.0096	–	K1a	0.0385	0.0380	T2b23	0.0192	–
H*	–	0.0633	K1a1b1a	0.0385	–	T2b5a1	0.0096	–
H1	0.0865	0.1772	K1a4a1a + 195	0.0096	0.0127	T2c1d	0.0481	0.0127
H2a2a	0.0385	0.0253	K1b1a1 + 199	0.0096	–	U1a1a	0.0481	–
H3	0.0288	0.0380	K1b1 + 16,093	–	0.0127	U2e1′2′3	–	0.0127
H4a1a	–	0.0253	K1c	0.0096	–	U2e1e	–	0.0127
H6a1	0.0096	0.0380	K2a5	–	0.0127	U2e2a2	–	0.0127
H11a	0.0096	0.0253	K2b1a1	0.0096	0.0506	U3	0.0192	–
HV0	–	0.0759	L2a1b + 143	–	0.0127	U3a	0.0096	–
HV0 + 195	0.0192	0.0380	L3d1b2	–	0.0127	U4b3	–	0.0127
HV15	–	0.0127	L3e2b + 152	0.0481	–	U5a2	0.0096	–
HV4a2a	–	0.0127	M1a1	0.0192	–	U5b1d2	0.0096	–
I	–	0.0127	M5a1	0.0192	–	U5b1f1a	0.0192	–
I1c1	0.0096	–	N1b1	–	0.0127	U5b2a2	–	0.0127
I2′3	–	0.0127	R0a + 60.1 T	0.2019	–	U5b2b1a1	–	0.0127
J1b1a1	–	0.0127	T	0.0288	0.0127	U5b2b3	–	0.0253
J1b1b	–	0.0127	T1a	0.0577	–	U5b3	0.0096	0.0253
J1c	–	0.0127	T1a1′3	0.0096	–	U6a	0.0096	–
J1c2o	0.0096	–	T2	0.0096	0.0127	V + 16,298	–	0.0127
J1d1	0.0096	–	T2a1b	–	0.0253	X2c	–	0.0127
J2a1a1	0.0288	–	T2b	0.0096	–	Unclassified	–	0.0127
J2b1a	0.0096	0.0127						

All in all, the mitochondrial haplogroup composition indicated that Chuetas are statistically different from their host population (Majorca) (*F*_ST_ p-values < 10^–5^). By means of the SAMPLING analyses, and also with the Behar et al.^22^ criterion, two haplogroups showed up as the founder lineages in the Chueta population: R0a + 60.1 T (~ 20%) and T1a (~ 6%).

The presence in Chuetas of haplogroups rarely found in neighbouring populations—L3eb + 152, U1a1a, and K1a1b1a (with frequencies of 5%, 5%, and 4%, respectively)—could also mean that they might have been present in the original Jewish Majorcan gene pool.

### Phylogenetic relation with other populations

#### Y-chromosome

The most frequent subclade of haplogroup R in Europe is R1b-M269, with frequencies ranging from 41 to 83%^50^. Ancient DNA studies carried out in recent years^51–54^ have shown that this lineage is associated with the spread of Steppe ancestry during the Bronze Age. In Iberia former Y-chromosome lineages were nearly completely replaced by haplogroup R1b-M269^53^, and a west-to-east gene flow from Iberia could have introduced these haplogroups into Western Mediterranean islands^54^. Majorca showed similar values (63%) to the rest of Iberian populations^19,46^, whereas the Chueta population (4%), had similar values to Middle Eastern and North African populations^55,56^. In Jewish populations, values range between 5% and 11.5%^19,57,58^, with the highest frequencies in Sephardim. These results support some degree of Iberian admixture in Sephardic Jews^19^ and important gene flow from the host population in Bragança Crypto-Jews (with an R1b-M269 frequency of 28%), as suggested by Nogueiro et al.^23^.

**Figure 3:**
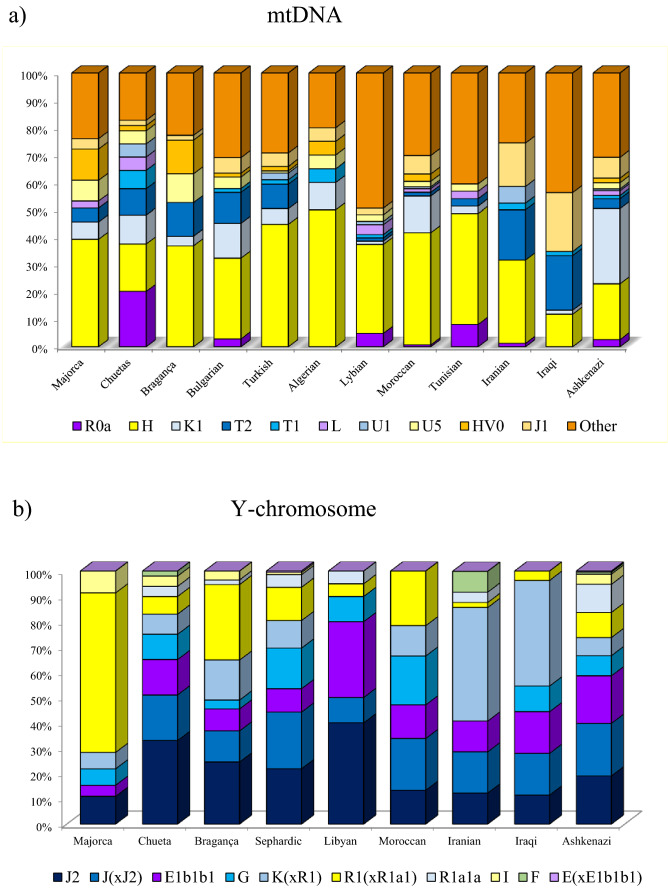
Haplogroup frequencies of mtDNA (**a**) and Y-chromosome (**b**) in Chuetas, Majorcans, and different populations with Jewish origin, based on data from the literature (Supplementary Table 5). Y-chromosome haplogroups are defined with the following SNPs: M172 (J2), 12f2a (J(xJ2)), M35 (E1b1b1), M201 (G), M9(xM173) K(xR1), M173(xM17) R1(xR1a1), M17 (R1a1), M170 (I), M213 (F), M1(xM35) E(xE1b1b1).

Haplogroup J-12f2a, thought to have originated in the Fertile Crescent^59^, shows an East to West gradient in Europe. Although the phylogeography of haplogroup J is complex, its radiation seems to be concentrated mainly in the Bronze Age, an essential period for the establishment of the modern European genetic pool^60^. It contains two major branches, one of them, J2-M172, has its high frequencies in the Levant and is the most frequent sub-haplogroup in Europe, mainly throughout the Mediterranean basin. It was long considered to have spread across Europe with the demic diffusion of Neolithic agriculturalists^61,62^, but ancient DNA studies contradict this hypothesis, since in early Middle Eastern farmers, and also in European Neolithic remains, haplogroup J2-M172 is only detected sporadically^63^. Signs of population movements from the East, mainly maritime, such as the Phoenicians^7,56,63^ have been linked to J2^56,60^ and also to the other main branch, J1-M267. This lineage has its maximum frequency in Arabia, but also high frequencies in the Middle East and in Jewish groups^58,64^. Some of its derived lines have purportedly been associated with Arabisation in North Africa^46,62^ while others have been implicated with different expansions of Middle Eastern populations through the Mediterranean Sea^56^. In most Jewish populations, J2-M172 reaches considerable frequencies^9,14,19,57^ (Fig. 3), but in Europeans it is about 10%. In the Chueta population, J2-M172 was the modal haplogroup with a frequency of 33%, while in its host population it was found in 10% of males. This value is similar to other Jewish populations and higher than the average Sephardic percentage (22%). Haplogroup J1-M267, not found in Majorca, was the second most frequent in Chuetas, with similar values (18%) to those in other Jews (Fig. 3)^14,19^.

Other Y-haplogroups with differential frequency between Chuetas and their host population were E1b-M78, Q1-P36.2, G-M201, and R1a1a-M17. E1b-M78 seems to have originated in north-eastern Africa and several lines of evidence suggest that some E1b-M78 derived lines have been involved in trans-Mediterranean migrations directly from Africa to Europe^65^. Chuetas showed a frequency for this haplogroup of 14%. In other populations with Jewish origin, it has been found to range from 3.5% (Bragança Crypto-Jews) to 15% (North African Jews)^19,23,57,58,61^; and in Middle East non-Jewish populations, from 10.0 to 17.0%^57^. Haplogroup Q1-P36.2 (xM346) is practically absent in Europe and Africa^19,66^. In Chuetas it showed a frequency of 8%, while in Jewish populations, percentages ranging from < 1% to 5% have been found^9,19,58,66^, with one branch typical of Ashkenazi Jews^66^. One of the main sub-branches of haplogroup G-M201, G2a, was the predominant male lineage in early European farmers, although the important Y-chromosome turnover in the Bronze Age nearly completed replaced it with the R1b lineage^52,53^. Nowadays haplogroup G-M201 is most common in Caucasus where the maximum frequencies are observed (> 70%), but it also occurs in the rest of the Middle East and south-western European countries at frequencies ranging from 5 to 15%^67^; while in North Africa it is far lower than in any European Mediterranean population^46^. In Jewish populations, Moroccan and Sephardic Jews have the highest values, 16% and 19%, respectively^14,23,57^ (Fig. 3). The existence of gene flow between Sephardim and Iberians (with frequencies reaching 5.0%) has been suggested^19,23^, although the direction of the introgression could not be determined. Lastly, the main subclade of the typical Eastern Europe R1a-M240^68^ is R1a1a-M17^69^, which is found in high percentages in Ashkenazim (up to 14.5%) and is also present in other non-Ashkenazi Jewish populations, but at a much lower frequency (4.4%)^9,70^. In Chuetas, R1a1a-M17 was found in 4% of the individuals analysed, but not in the Majorcan samples. It could be interesting to assess whether the Chueta R1a1a chromosomes belong to the subclade shared by Ashkenazim and other Middle Eastern populations (M582), but absent in Eastern Europeans^70^.

All in all, the Y-chromosome haplogroup profile in Chuetas is clearly dissimilar from their neighbouring population, Majorca, and quite similar to the haplogroup frequencies found in Sephardic and other Jewish populations, evidencing a considerable frequency of J2 + J1 haplogroups and low values of R1b (Fig. 3). Therefore, it seems likely that the differential presence in the Chueta population of both haplogroups (and also of E1b-M78, Q1-P36.2, G-M201, and R1a1a-M17) results from their well-known historical Jewish origin and/or by admixture with other Jewish groups, especially with North Africans, due to commercial contact between both communities^71^.

**Figure 4:**
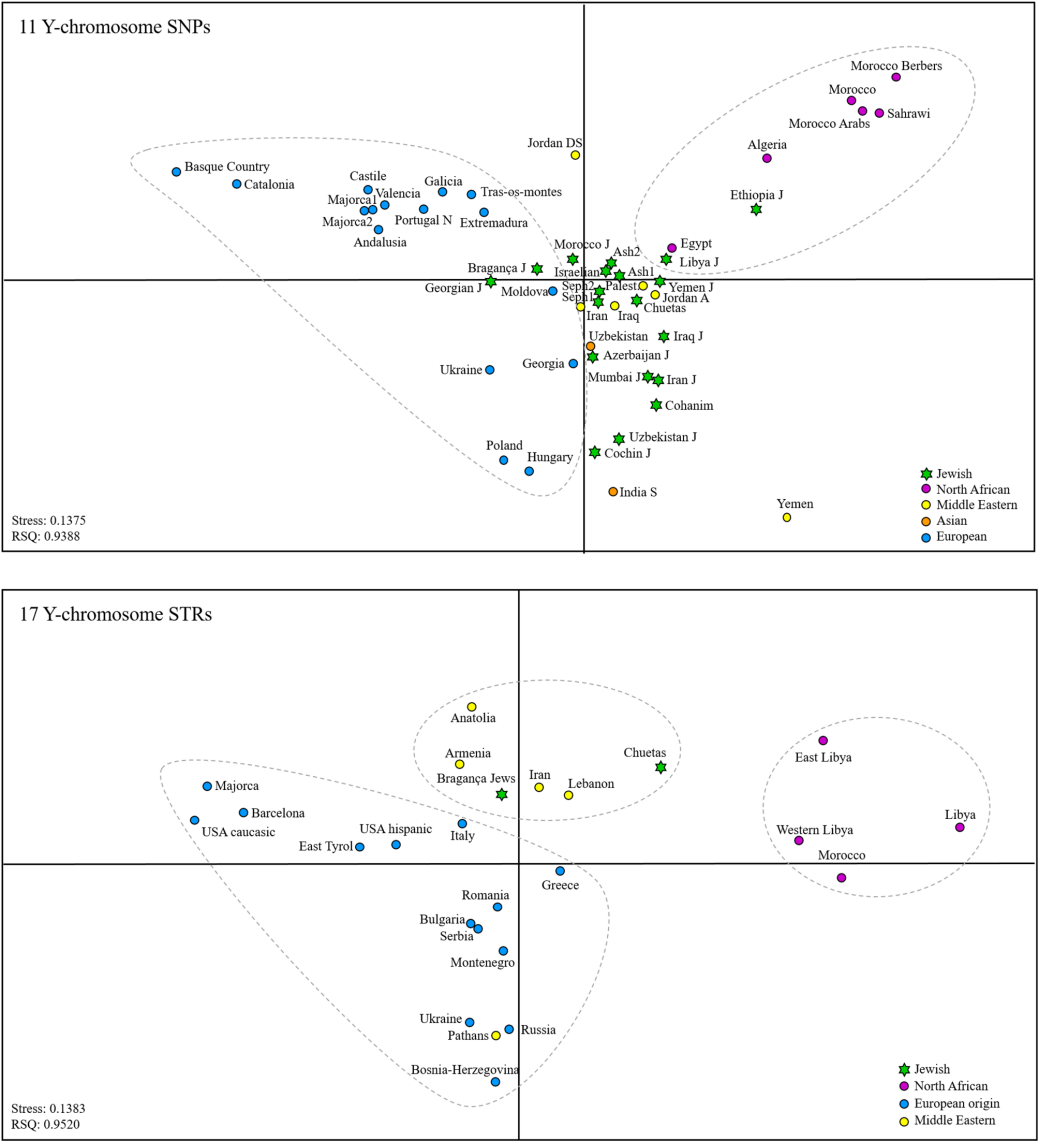
Multidimensional Scaling plot performed with (**a**) 11 SNPs: M1(xM35) E(xE1b1b1), M35 (E1b1b1), M213 (F), M201 (G), M170 (I), 12f2a (J(xJ2)), M172 (J2), M9(xM173) K(xR1), M173(xM17) R1(xR1a1), M17 (R1a1) and a final category for other haplogroups not included in these 10 SNPs and (**b**) Y-filer STRs. Circles defining each population are coloured following the legend code. Jewish populations are labelled with a Star of David. Populations and references are in Supplementary Table 5.

A comparison with available populations in the literature, based on both STRs and SNPs, (Fig. 4) positioned Chuetas with other Jewish and Middle Eastern populations, far from their geographical neighbours, evidencing that Chuetas maintain, in male lineages, substantial relics of their Jewish ancestry.

#### Mitochondrial DNA

**Figure 5:**
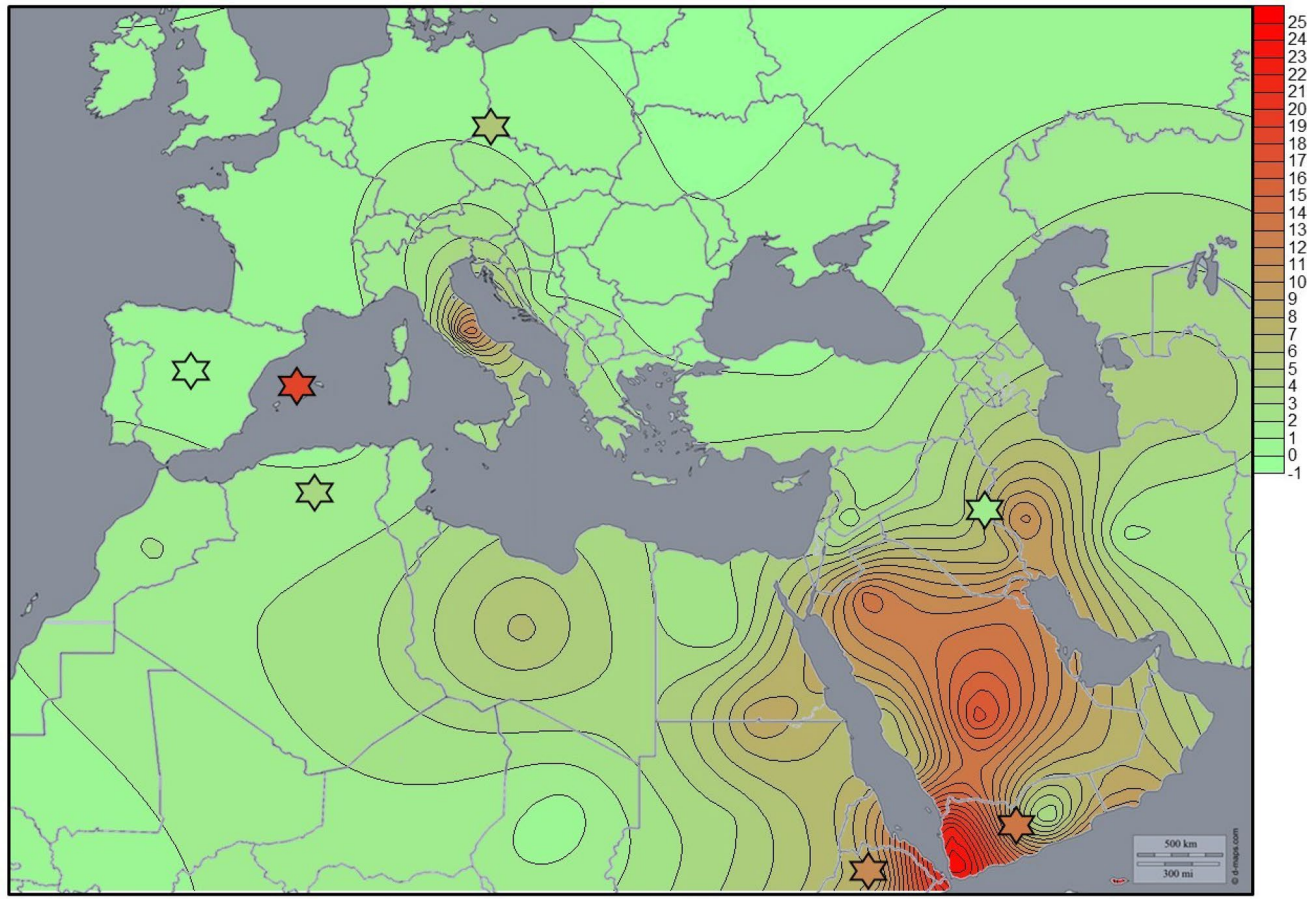
Isofrequency map of haplogroup R0a based on data from the literature (Supplementary Table 5) generated by Surfer v.8 (Golden Software Inc., Golden, Colorado) using a base map image (http: d-maps.com). Jewish populations are indicated with a Star of David.

The origin in terms of location and timescale of the Chuetas' modal haplogroup R0a has been under debate in recent years due to the geographic distribution of its frequencies^72–74^. As shown in Fig. 5, this haplogroup is practically absent in Europe (0–2%), although some exceptions are found, such as Cappadocia village in Italy (14.6%)^75^. The highest frequencies of haplogroup R0a are found in the Arabian Peninsula and the Horn of Africa, reaching values as high as ~ 25% in Soqotra Island in Yemen^73^. Frequencies in Jewish groups^8,22,73,76^ on the whole show similar frequencies to their host populations (Fig. 5), but not in the case of Chuetas, who present a frequency of R0a of 20.2% while it is absent in their host population. The first dating of the haplogroup (~ 19 Kya) suggested an Arabian origin^72^. Later studies dated the haplogroup earlier, ~ 22.5 Kya^73^ and ~ 30 Kya^74^. Both studies discuss whether the origin could be in the Horn of Africa or the Arabian Peninsula. Phylogeographic differences in the regional distribution of R0a and the fact that the most ancient reservoir of R0a variation is found in Arabia led the authors to conclude an Arabian origin of the haplogroup. Two main branches characterise this haplogroup: R0a1 (~ 26 Kya) and R0a2′3 (~ 21 Kya)*.* R0a2′3 is defined by the insertion 60.1 T and most of the Jewish populations where information is available show this branch. Only Yemenite Jews show both^22^.

**Figure 6:**
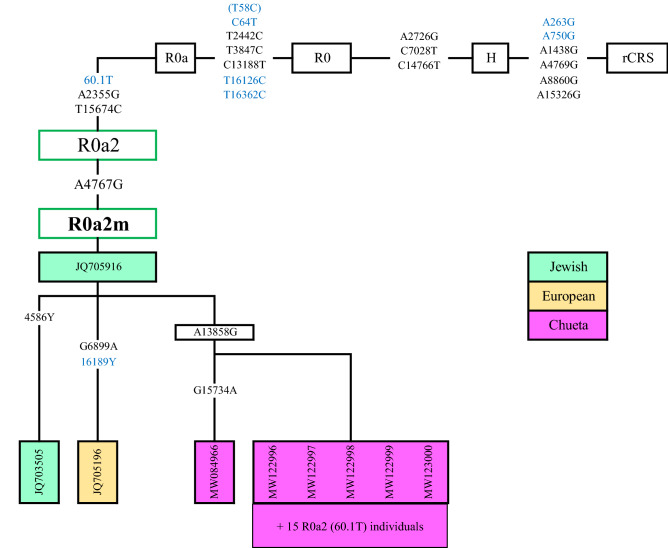
Phylogenetic tree of the completed mtDNA sequences belonging to haplogroup R0a2m. Mutations relative to the revised Cambridge Reference Sequence (rCRS) (NC_012920) are shown. Mutations coloured in blue correspond to the D-loop. The origin of the samples in the haplogroup are labelled in colours. In green, two Jewish samples from the Czech Republic and Ukraine (JQ705916 and JQ705196); and in yellow, one sample from Poland with unknown ethnicity (JQ703505). Finally, Chueta samples, with their corresponding GenBank accession numbers, are labelled in pink. The remaining 15 R0a + 60.1 T Chueta samples were found to be R0a2m (A4767G). Presence of the A13858G mutation was confirmed in all, whereas the G15734A mutation remained as a private variant of the MW084966 sample.

Taking into account the high prevalence of this haplogroup in the Chueta population, it was considered important to delve into the phylogeny of their possible main maternal founder. Thus, the complete mtDNA genome was obtained for 6 out of the 21 R0a + 60.1 T samples, which classified the Chueta samples as R0a2m. The R0a2m branch, dated by Gandini et al.^74^ to ~ 1.4 Kya, is found in just three samples in the literature, two of them from Jewish origin and the other with unknown ethnicity. In Fig. 6, it can be seen that all six complete Chueta molecules share an additional mutation (A13858G), whereas one sample has a G15734A private mutation. In all the rest of R0a + 60.1 T Chueta samples, we have checked the defining mutation of the R0a2m branch (A4767G). Besides, the two specific Chueta positions have also been examined, revealing that A13858G was present in all 21 individuals, suggesting a new R0a2m sub-branch in this population, while the G15734A mutation remained as a private variant.

The time of the appearance of haplogroup JT can be estimated at ~ 58 Kya, before the settlement of the Fertile Crescent. It has been suggested that haplogroups J and T diverged during the settlement ~ 40 Kya and ~ 30 Kya, respectively^77^. Haplogroup J has higher frequencies in the Middle East and Arabia than in Europe (13–20% vs. 9%), while haplogroup T shows the opposite behaviour (10% in Europe and 8% in the Middle East)^77,78^. No significant differences were found between modern and ancient Majorcan populations regarding haplogroup frequencies since at least the Bronze Age^79^. T1a (5.8%) is considered one of the founders of the Chueta population, and originated in the Near East ~ 17 Kya, although most of its sub-branches seem to be European^77^.

Regarding the other mtDNA haplogroups with differential frequencies between Chuetas and their host population, Haplogroup U is the second most frequent in modern Europeans and was predominant in pre-agricultural Europe^80^. The U1a sub-haplogroup is dated at ~ 13–15 Kya and is present in Southwest and South Asia, the Caucasus, and Europe. Five U1a1a1 samples were found in the Chueta population, whereas no U1 sub-haplogroup was observed in the host population. Haplogroup K origin has been dated to ~ 36 Kya and, although the place of origin is still under discussion^81,82^, a Levantine origin seems the most likely. Sub-haplogroup K1a1b1a (dated to ~ 4.4 Kya^82^), found in four Chueta samples, is a founder lineage in Ashkenazim^81^ and also present in Sephardic communities^22^, but not in non-European Jews, which can be seen as evidence of its European origin^82^.

Haplogroup L3e is widespread in Africa but practically absent in Eurasia (except in neighbouring areas due to genetic exchange). Its origin is situated in Central or Eastern Africa about 46 Kya^83^ and one of its most frequent lineages in West-Central Africa is L3e2b (7%)^84^. Five Chueta samples showed haplogroup L3e2b, while in the host population just one sample presented one African haplogroup L3, but belonging to another subclade, L3d.

Founding lineages in Chuetas, R0a + 60.1 T (~ 20%) and T1a (~ 6%), are different from those of other Sephardic populations, which also show important dissimilarity between each other. For instance, while HV0b is found as a founder in the Portuguese Crypto-Jewish communities from Belmonte and Bragança^22–24^, this haplogroup is absent or very uncommon in Chuetas and other Sephardic groups; additionally, haplogroup K1a1b1a, which is a founder (8.5%) in the Iberian Exile Jewish community from Bulgaria, has a lower frequency in other populations with Sephardic origin (4% in Chuetas and 0.8% in Turkish Jews)^22^.

## Conclusions

Genetic diversity in both paternal and maternal lineages in the Chueta population was higher than expected for a small, endogamous population. Comparable high diversity values were observed in the Portuguese Crypto-Jewish communities of Bragança^23,24^. These results reveal that demographic processes more complex than the loss of genetic diversity expected under conditions of extreme inbreeding and drift, have shaped the gene pool of both isolated populations. Ongoing data from recombinant markers, together with classical genealogical studies, will help to explain what mating strategies were undertaken by these communities to avoid the expected reduction of diversity, and also whether other factors, such as high heterogeneity in founder populations, could have contributed to the diversity observed.

The composition of the Chuetas’ uniparentally transmitted lineages indicates a remarkable signature of Middle Eastern ancestry. In recent years, archaeogenetic research has shed light on the history of European and Middle Eastern populations, revealing a greater degree of population movements and interactions in the past than previously considered. In this context, from the analysis of current populations, it is difficult to infer what the genetic profile of the parental populations that gave rise to the Chuetas was exactly, and therefore precise inferences of the original source of the haplogroups found in this population, since there are no reasons to assume or assess the degree of genetic continuity. However, the most reasonable explanation for the differences found between Chuetas and their host population seems to be the Jewish origin of the Chuetas, considering the unquestionable historical evidence relating them with the Jewish populations who settled in Majorca long ago in the past. Therefore, our results would confirm that Chuetas have kept not only the cultural memory of their Jewish origin over centuries, but also a substantial degree of ancestral genetic signature.

In terms of paternal lineages, the results show that most Jewish communities are more similar to each other and to Middle Eastern populations than to their host populations. The Chueta population has the same behaviour, which can be observed by the high prevalence of haplogroups J2-M172 and J1-M267, and the lack of R1b-M269. Haplogroup distribution in Chuetas is very similar to other Sephardic communities, although in their gene pool there might be signatures of other Jewish communities’ contribution, such as North African and Ashkenazim, which can be inferred from the presence of haplogroups such as E1b1b1a1-M78, Q1-P36.2, and R1a1a-M17.

The hallmark in the maternal gene pool in Chuetas is the presence of a new sub-branching of the rare Middle Eastern haplogroup R0a + 60.1 T as their modal haplogroup, and the low frequency of H. The presence of other haplogroups found in Jewish/Middle Eastern populations (K1a1b1a and U1a1a1) is also noteworthy. Current Jewish populations do not usually share modal maternal lineages, unlike the situation on the paternal side; not even populations with a supposed common ancestry, such as the Sephardic branch^8,22^. Various scenarios could explain this dissimilarity found in maternal founder lineages in distinct current populations with Sephardic origin: differences in gene flow from and admixture with other populations resulting from the contrasting history of each community; a lack of homogeneity in maternal lineages of the original Sephardic groups that settled in different areas of the Iberian Peninsula (and Balearic Islands); or genetic drift in the current populations, resulting in a lack of lineages that do not allow us to infer the original mtDNA composition of the Sephardic Jews that lived in Spain and Portugal in the middle ages from the surviving lines. Tests based on genome-wide data that enable a greater genetic resolution, such as analysis of Runs of Homozygosity (ROH), together with ancient DNA analysis, could help to solve this question.

## Supplementary information

Supplementary Information.
