# Supplementary material for: Middle eastern genetic legacy in the paternal and maternal gene pools of Chuetas
Source: Sci Rep. 2020 Dec 8;10:21428. doi: 10.1038/s41598-020-78487-9 (PMC7722846; doi:10.1038/s41598-020-78487-9)
Supplement: Supplementary file 1 — Supplementary Information. [file 41598_2020_78487_MOESM1_ESM.pdf]

MIDDLE EASTERN GENETIC LEGACY IN THE PATERNAL AND MATERNAL GENE POOLS OF CHUETAS

Ferragut JF[a]; Ramon C[a]; Castro JA[a]; Amorim A[b,c,d]; Alvarez L[b,c,e]; Picornell A[a]

[a]Institut Universitari d'Investigació en Ciències de la Salut (IUNICS) i Laboratori de Genètica, Departament de Biologia, Universitat de les Illes Balears, Carretera de Valldemossa, km 7.5, 07122, Palma de Mallorca, Illes Balears, Spain

[b]i3S - Instituto de Investigação e Inovação em Saúde, Universidade do Porto, Rua Alfredo Allen, 208, 4200-135 Porto, Portugal

[c]IPATIMUP - Instituto de Patologia e Imunologia Molecular da Universidade do Porto, Rua Júlio Amaral de Carvalho, 45, 4200-135 Porto, Portugal

[d]Faculdade de Ciências da Universidade do Porto, Rua do Campo Alegre, s/n, 4169-007 Porto, Portugal

[e]TellmeGen™, Calle Arquitecto Mora, 5 – 4, 46010, Valencia, España

Supplementary Table 1. Allele frequencies for 17 Y-chromosome STR loci in Chueta (CHU) and Majorcan (MAJ) populations.

Allele	DYS456		DYS389I		DYS390		DYS389II		DYS458		DYS19		DYS393		DYS391		DYS439		DYS635		DYS392		Y GATA H4		DYS437		DYS438		DYS448	
**N**	CHU 100	MAJ 46	CHU 100	MAJ 46	CHU 100	MAJ 46	CHU 100	MAJ 46	CHU 100	MAJ 46	CHU 99	MAJ 46	CHU 100	MAJ 46	CHU 100	MAJ 46	CHU 100	MAJ 46	CHU 100	MAJ 46	CHU 100	MAJ 46	CHU 100	MAJ 46	CHU 100	MAJ 46	CHU 100	MAJ 46	CHU 100	MAJ 46
**8**																								0.022						
**9**															0.180	0.087											0.320	0.130		
**10**			0.040										0.010		0.720	0.543	0.050	0.022					0.090	0.022			0.490	0.196		
**11**													0.010		0.090	0.370	0.400	0.283			0.840	0.261	0.510	0.413			0.150	0.043		
**12**	0.010		0.030	0.239									0.420	0.196	0.010		0.430	0.543			0.010	0.065	0.320	0.500			0.030	0.609		
**13**	0.090	0.021	0.880	0.521							0.172	0.043	0.460	0.696			0.120	0.130			0.070	0.630	0.070	0.043			0.010	0.022		
**14**	0.140	0.043	0.040	0.239					0.110		0.465	0.674	0.090	0.109				0.022				0.043	0.010		0.630	0.370				
**15**	0.370	0.587	0.010						0.090	0.043	0.253	0.261													0.260	0.500				
**16**	0.290	0.326							0.130	0.283	0.071	0.022									0.060				0.110	0.130				
**17**	0.100	0.021							0.420	0.326	0.040										0.020									
**17.2**									0.100																					
**18**									0.050	0.326																				0.130
**18.2**									0.060																					
**19**									0.020	0.022																			0.280	0.587
**19.2**									0.020																					
**20**																			0.080	0.043									0.420	0.130
**21**																			0.510	0.196									0.300	0.065
**22**					0.220	0.109													0.270	0.109										0.087
**23**					0.540	0.217													0.140	0.500										
**24**					0.190	0.565														0.109										
**25**					0.050	0.087														0.043										
**26**						0.022																								
**27**							0.040																							
**28**							0.150	0.174																						
**29**							0.190	0.478																						
**30**							0.320	0.326																						
**31**							0.220																							
**32**							0.080	0.022																						
**NA**	6	5	5	3	4	5	6	4	9	5	5	4	5	3	4	3	4	5	4	6	5	4	5	5	3	3	5	5	3	5
**D**	0.749	0.558	0.224	0.627	0.628	0.627	0.791	0.648	0.777	0.721	0.691	0.486	0.600	0.476	0.445	0.573	0.645	0.620	0.647	0.700	0.288	0.540	0.631	0.589	0.529	0.610	0.640	0.585	0.662	0.623

Alleles	DYS385	
**N**	CHU 100	MAJ 46
**13-18**	0.230	
**13-16**	0.110	0.065
**14-16**	0.240	0.028
**11-14**	0.050	0.391
**16-16**	0.120	
**13-13**	0.050	
**12-12**	0.050	0.022
**11-15**	0.030	0.087
**13-15**	0.010	
**11-12**	0.010	
**14-14**	0.020	0.022
**19-19**	0.010	
**13-14**	0.010	0.043
**15-15**	0.010	0.022
**15-16**	0.010	0.022
**18-19**	0.030	
**12-13**	0.010	
**12-14**		0.043
**14-20**		0.022
**11-13**		0.087
**13-17**		0.065
**18-21**		0.022
**10-14**		0.022
**11-16**		0.022
**14-18**		0.022
**NA**	17	17
**D**	0.861	0.833

N: Number of individuals, NA: number of different alleles, D: Gene Diversity

Supplementary Table 2. Y-chromosome STR haplotype (Ht) distribution found in Chueta (CHU) (YHRD: YA004701) and Majorcan (MAJ) populations (YHRD: YA004702).

Ht	Haplogroup	CHU	MAJ	DYS456	DYS389I	DYS390	DYS389II	DYS458	DYS19	DYS385a	DYS385b	DYS393	DYS391	DYS439	DYS635	DYS392	Y GATA H4	DYS437	DYS438	DYS448
**01**	E1b1b1a1-M78	1	-	15	13	23	29	17	14	18	19	13	10	13	20	11	12	14	10	21
**02**	E1b1b1a1-M78	1	-	15	13	23	30	17	14	18	19	13	10	13	20	11	12	14	10	21
**03**	E1b1b1a1-M78	1	-	15	13	23	30	17	14	18	19	13	11	13	20	11	12	14	10	21
**04**	E1b1b1a1-M78	1	-	16	13	24	30	16	13	16	16	13	10	11	22	11	12	14	10	20
**05**	E1b1b1a1-M78	1	-	17	13	24	30	16	13	15	16	13	10	11	22	11	12	14	10	20
**06**	E1b1b1a1-M78	1	-	17	15	24	32	16	13	16	16	13	10	11	23	11	12	14	10	20
**07**	E1b1b1a1-M78	1	-	15	13	23	30	17	14	19	19	13	10	12	20	11	12	14	10	21
**08**	E1b1b1a1-M78	7	-	17	13	24	30	16	13	16	16	13	10	11	22	11	12	14	10	20
**09**	E1b1b1a1-M78	-	1	15	14	24	32	18	14	18	21	12	10	12	20	13	13	14	10	20
**10**	E1b1b1b-M81	-	1	15	14	24	30	18	13	13	16	13	9	10	21	11	11	14	10	20
**11**	F-M213	2	-	15	14	22	30	18	15	16	16	12	10	11	20	11	12	14	9	20
**12**	G-M201	1	-	13	13	22	32	17	15	13	13	14	9	11	22	11	11	16	10	21
**13**	G-M201	1	-	12	13	22	32	17	15	13	13	14	9	11	22	11	11	16	10	21
**14**	G-M201	1	-	13	13	22	30	17	15	13	13	14	9	11	22	11	11	16	10	21
**15**	G-M201	1	-	13	13	22	32	17	15	13	13	13	9	11	22	11	11	16	10	21
**16**	G-M201	2	-	13	13	22	32	17	15	13	13	14	9	11	23	11	11	16	10	21
**17**	G-M201	1	-	13	13	22	32	18	15	13	13	14	9	11	22	11	11	16	10	21
**18**	G-M201	1	-	14	12	22	29	17	15	14	14	14	10	10	21	11	12	15	9	20
**19**	G-M201	1	-	15	12	22	29	16	15	14	14	14	10	12	20	11	12	16	10	21
**20**	G-M201	1	-	15	12	22	29	18	15	14	14	10	10	11	20	11	12	16	10	21
**21**	G-M201	-	1	14	12	22	29	17	15	15	15	14	10	12	20	11	11	16	11	21
**22**	G-M201	-	1	15	12	22	28	16	15	15	16	12	10	12	21	11	11	16	10	22
**23**	G-M201	-	1	15	12	23	28	17	15	13	14	14	10	12	21	12	11	16	10	22
**24**	I-M170	1	-	15	14	23	32	16	17	15	15	14	10	12	21	12	11	14	10	20
**25**	I-M170	-	1	13	12	26	28	16	15	13	17	13	10	11	21	11	10	15	10	19
**26**	I-M170	-	1	14	12	22	28	15	14	13	14	13	10	11	21	11	11	15	10	20
**27**	I-M170	-	2	15	14	23	30	18	15	11	13	13	11	11	21	11	11	16	10	20
**28**	I2a1a1-M26	1	-	14	13	23	28	17	17	11	12	13	10	11	21	11	13	15	10	21
**29**	I2a1a1-M26	2	-	14	13	23	29	17	17	12	12	13	10	11	21	11	13	15	10	21
**30**	J1-DYS458.2	1	-	16	13	23	30	17.2	14	13	18	12	10	12	21	11	11	14	10	20
**31**	J1-DYS458.2	1	-	16	13	23	31	17.2	14	13	18	11	10	12	21	11	11	14	10	20
**32**	J1-DYS458.2	7	-	16	13	23	31	17.2	14	13	18	12	10	12	21	11	11	14	10	20
**33**	J1-DYS458.2	1	-	16	13	23	31	17.2	14	13	18	12	10	12	21	11	11	14	10	21
**34**	J1-DYS458.2	6	-	16	13	23	31	18.2	14	13	18	12	10	13	21	11	11	14	10	20

**35**	J1-DYS458.2	2	-	16	13	23	31	19.2	14	13	18	12	10	13	21	11	11	14	10	20
**36**	J2-M172	1	-	15	13	22	31	15	16	13	14	12	10	11	23	11	10	15	9	21
**37**	J2-M172	2	-	15	13	23	30	17	14	13	18	12	10	11	22	11	11	16	9	20
**38**	J2-M172	9	-	14	13	23	28	14	15	13	16	13	9	12	21	11	12	14	9	21
**39**	J2-M172	1	-	14	13	23	28	14	16	13	16	13	9	12	21	11	12	14	9	21
**40**	J2-M172	1	-	15	13	22	31	15	16	13	15	12	10	11	21	11	12	14	9	21
**41**	J2-M172	1	-	15	13	23	28	14	15	13	16	13	9	12	21	11	12	14	9	21
**42**	J2-M172	1	-	15	13	23	29	17	14	14	16	12	10	11	21	11	11	15	9	19
**43**	J2-M172	12	-	15	13	23	30	17	14	14	16	12	10	11	21	11	11	15	9	19
**44**	J2-M172	1	-	15	13	23	30	17	14	14	16	12	10	12	21	11	11	15	9	19
**45**	J2-M172	1	-	16	10	24	27	17	14	13	18	12	10	12	21	11	11	15	10	20
**46**	J2-M172	1	-	16	10	24	27	17	14	13	18	12	10	12	22	11	11	15	10	20
**47**	J2-M172	1	-	16	10	24	27	18	14	13	18	12	10	12	22	11	11	15	10	20
**48**	J2-M172	1	-	16	10	25	27	17	14	13	18	12	10	12	22	11	11	15	10	20
**49**	J2-M172	-	1	15	14	24	30	16	14	14	18	12	10	11	22	11	11	16	10	20
**50**	J2-M172	-	1	16	12	22	30	18	14	13	17	12	9	11	22	11	12	15	9	22
**51**	J2-M172	-	1	16	12	22	30	18	14	13	17	12	10	11	22	11	12	15	9	22
**52**	J2-M172	-	1	16	13	23	29	16	15	13	16	12	9	12	22	11	12	14	9	21
**53**	J2-M172	-	1	16	13	23	29	17	15	13	16	12	9	12	22	11	12	14	9	21
**54**	P-92R7	-	1	15	14	23	30	16	14	14	20	13	10	11	25	12	11	15	11	19
**55**	Q1-P36.2	1	-	15	13	22	28	17	13	14	16	13	10	12	22	16	10	14	11	19
**56**	Q1-P36.2	4	-	15	13	22	29	17	13	14	16	13	10	12	22	16	10	14	11	19
**57**	Q1-P36.2	2	-	15	13	22	29	17	13	14	16	13	10	12	22	17	10	14	11	19
**58**	Q1-P36.2	1	-	15	13	22	29	17	?	14	16	13	10	12	22	16	10	14	11	19
**59**	R1a1a-M17	1	-	16	13	25	30	15	16	11	14	13	11	10	23	11	14	14	11	20
**60**	R1a1a-M17	2	-	16	13	25	31	15	16	11	14	13	11	10	23	11	13	14	11	20
**61**	R1a1a-M17	1	-	16	13	25	31	15	16	11	15	13	11	10	23	11	13	14	11	20
**62**	R1b1a2a-M18	2	-	13	13	24	28	15	15	12	12	13	11	12	23	13	11	14	11	19
**63**	R1b1a2a-M18	1	-	13	14	24	29	15	15	12	12	13	11	12	23	13	11	14	11	19
**64**	R1b1a1a2a-L23	-	1	15	13	24	29	16	15	11	15	13	11	11	23	13	13	15	12	19
**65**	R1b1a1a2a1a1-U106	-	1	15	13	23	29	18	14	11	14	13	10	12	23	14	12	15	12	19
**66**	R1b1a1a2a1a2-S116	1	-	16	13	23	29	19	14	11	14	13	12	12	21	13	12	15	12	19
**67**	R1b1a1a2a1a2-S116	1	-	16	13	24	29	17	14	11	14	12	10	12	23	13	11	14	12	19
**68**	R1b1a1a2a1a2-S116	-	1	15	12	24	28	16	14	11	15	13	10	12	23	13	12	15	12	19
**69**	R1b1a1a2a1a2-S116	-	1	15	12	25	28	17	14	11	13	13	10	14	24	13	11	15	12	19
**70**	R1b1a1a2a1a2-S116	-	1	15	12	25	28	17	14	11	13	13	11	13	24	13	11	15	12	19
**71**	R1b1a1a2a1a2-S116	-	1	15	13	24	29	16	14	11	14	13	11	12	23	13	11	14	12	18
**72**	R1b1a1a2a1a2-S116	-	1	15	13	24	29	16	14	11	15	13	11	12	23	13	12	15	12	19

**73**	R1b1a1a2a1a2-S116	-	1	15	13	24	29	17	14	11	14	14	10	12	25	13	11	14	12	18
**74**	R1b1a1a2a1a2-S116	-	1	15	13	24	29	18	14	11	14	13	10	12	23	13	12	14	12	19
**75**	R1b1a1a2a1a2-S116	-	1	15	13	24	30	16	14	11	14	13	11	13	23	13	12	15	12	19
**76**	R1b1a1a2a1a2-S116	-	1	15	13	24	30	16	16	11	14	13	11	12	24	13	12	15	12	19
**77**	R1b1a1a2a1a2-S116	-	1	15	13	24	30	18	14	11	14	12	10	11	23	13	11	14	12	18
**78**	R1b1a1a2a1a2-S116	-	1	15	14	24	29	17	14	11	14	13	10	12	23	13	11	14	12	18
**79**	R1b1a1a2a1a2-S116	-	1	15	14	24	30	17	14	11	15	13	11	12	23	13	12	15	12	19
**80**	R1b1a1a2a1a2-S116	-	1	16	12	25	28	17	14	11	14	13	10	12	23	13	12	15	13	19
**81**	R1b1a1a2a1a2-S116	-	1	16	13	23	29	17	15	10	14	13	10	12	23	13	12	14	12	19
**82**	R1b1a1a2a1a2-S116	-	1	16	13	24	29	16	14	11	14	12	11	12	23	13	11	14	12	19
**83**	R1b1a1a2a1a2-S116	-	1	16	13	24	29	17	14	12	14	13	11	13	23	13	11	14	12	18
**84**	R1b1a1a2a1a2-S116	-	1	16	13	24	29	18	14	11	14	13	11	12	23	13	12	15	12	19
**85**	R1b1a1a2a1a2-S116	-	1	16	13	24	29	19	14	11	14	13	11	13	23	13	12	15	12	19
**86**	R1b1a1a2a1a2-S116	-	1	16	13	24	30	18	14	11	14	14	10	12	23	12	12	15	12	19
**87**	R1b1a1a2a1a2-S116	-	1	16	14	24	30	17	14	11	16	13	11	13	23	13	12	14	12	18
**88**	R1b1a1a2a1a2-S116	-	1	17	13	24	29	18	14	11	14	13	11	11	23	13	12	14	12	19
**89**	R1b1a1a2a1a2b-U152	1		15	13	24	29	16	14	11	15	13	10	13	23	13	13	15	13	20
**90**	R1b1a1a2a1a2b-U152	-	1	15	13	25	29	18	14	12	14	13	10	12	23	13	12	14	12	19
**91**	R1b1a1a2a1a2b-U152	-	1	15	14	24	30	18	14	12	12	14	11	12	24	13	12	15	12	19
**92**	R1b1a1a2a1a2b-U152	-	2	16	13	24	29	17	14	11	14	13	10	12	23	13	12	15	12	19
**93**	R1b1a1a2a1a2c1-M529	1	-	17	13	24	29	19	14	12	13	13	11	12	23	13	11	15	12	19
**94**	R1b1a1a2a1a2c1-M529	-	1	15	13	24	29	17	15	11	14	13	10	13	23	13	12	15	12	19
**95**	R1b1a1a2a1a2c1-M529	-	1	16	13	24	29	16	14	11	14	13	11	11	24	13	8	15	12	19
**96**	T-M70	-	1	15	13	23	29	18	15	14	16	13	10	11	21	13	11	14	9	19
**97**	T-M70	-	1	15	14	23	30	15	13	14	14	13	10	12	21	14	11	14	9	19

Supplementary Table 3. Mitochondrial D-loop haplotypes and haplogroups of the 104 Chueta samples (EMPOP*: EMP00672), classified according to HaploGrep2 (Build 17, PhyloTree).

Sample	Haplogroup	Haplotypes																	
CHU2	U1a1a1	16182C	16183C	16189C	16249C	73G	263G	285T	309.1C	309.2C	315.1C	385G	d523	d524	573.1C	573.2C	573.3C		
CHU3	H1a3	16051G	16162G	16519C	73G	263G	315.1C	534T											
CHU4	U5b1f1a	16192T	16270T	16319A	73G	150T	263G	315.1C	533G										
CHU10	H1bo	16189C	16519C	263G	267C	315.1C	485C												
CHU11	U5b3	16192T	16270T	16304C	16526A	73G	150A	228A	263G	309.1C	315.1C								
CHU12	H1+152	152C	263G	309.1C	309.2C	315.1C	466C												
CHU15	K1a1b1a	16223T	16224C	16234T	16311C	16519C	73G	114T	263G	315.1C	497T								
CHU16	K1a	16129A	16224C	16256T	16311C	16519C	73G	263G	315.1C	497T									
CHU17	H66a	16172C	16519C	263G	315.1C														
CHU19	H2a2a	16519C	263G	315.1C															
CHU21	T1a	16126C	16163G	16186T	16189C	16294T	16298C	16319A	16519C	73G	263G	309.1C	315.1C						
CHU22	H66a	16172C	16519C	263G	315.1C														
CHU23	M5a1	16129A	16223T	16291T	16298C	16519C	73G	263G	315.1C	489C	524.1A	524.2C							
CHU24	R0a+60.1T	16126C	16362C	16519C	58C	60.1T	64T	263G	309.1C	315.1C									
CHU25	K1c	16224C	16311C	16519C	73G	146C	152C	263G	309.1C	315.1C	d498								
CHU26	R0a+60.1T	16126C	16362C	16519C	58C	60.1T	64T	263G	309.1C	315.1C									
CHU27	H1+152	152C	263G	309.1C	309.2C	315.1C	466C												
CHU28	R0a+60.1T	16126C	16362C	16519C	58C	60.1T	64T	263G	309.1C	315.1C									
CHU29	U3	16343G	73G	150T	263G	309.1C	315.1C												
CHU30	R0a+60.1T	16126C	16362C	16519C	58C	60.1T	64T	263G	309.1C	315.1C									
CHU31	R0a+60.1T	16126C	16362C	16519C	58C	60.1T	64T	263G	309.1C	315.1C									
CHU32	R0a+60.1T	16126C	16362C	16519C	58C	60.1T	64T	263G	309.1C	315.1C									
CHU33	H2a2a	16519C	263G	309.1C	315.1C														
CHU34	T2b	16126C	16294T	16296T	16304C	16519C	73G	263G	309.1C	309.2C	315.1C								
CHU35	T2c1d	16126C	16292T	16294T	16519C	73G	146C	263G	279C	309.1C	315.1C								
CHU36	H	16223T	16519C	263G	315.1C														
CHU37	J1d1	16069T	16126C	16193T	16300G	73G	152C	195C	263G	295T	309.1C	315.1C	462T	489C					
CHU38	H2a2a	16519C	263G	309.1C	315.1C	d523	d524												
CHU39	H1j8	16129A	16240G	16519C	152C	185A	263G	309.1C	315.1C										
CHU40	U6a	16092C	16172C	16219G	16278T	73G	263G	315.1C											
CHU42	T	16126C	16294T	16304C	16519C	73G	263G	309.1C	309.2C	315.1C									
CHU43	H11a2	16092C	16169T	16293G	16298C	16311C	263G	315.1C											
CHU45	T2c1d	16126C	16292T	16294T	16519C	73G	146C	263G	279C	309.1C	309.2C	315.1C							
CHU46	R0a+60.1T	16126C	16362C	16519C	58C	60.1T	64T	263G	309.1C	315.1C									
CHU47	R0a+60.1T	16126C	16362C	16519C	58C	60.1T	64T	263G	309.1C	315.1C									
CHU48	I1c1	16129A	16223T	16264T	16270T	16311C	16319A	16362C	16391A	16519C	73G	199C	204C	250C	263G	309.1C	315.1C	455.1T	573.1C
CHU49	M5a1	16129A	16223T	16291T	16298C	16519C	73G	263G	315.1C	489C	524.1A	524.2C							
CHU52	M1a1	16129A	16183C	16189C	16193.1C	16223T	16249C	16311C	16359C	16519C	73G	195C	263G	315.1C	489C				

CHU53	J2a1a1	16069T	16126C	16145A	16231C	16261T	73G	150T	152C	195C	203A	215G	263G	295T	315.1C	319C	489C	513A
CHU54	K1a	16129A	16224C	16256T	16311C	16519C	73G	263G	315.1C	497T								
CHU55	J2a1a1	16069T	16126C	16145A	16231C	16261T	73G	150T	152C	195C	203A	215G	263G	295T	315.1C	319C	489C	513A
CHU56	R0a+60.1T	16126C	16362C	16519C	58C	60.1T	64T	263G	309.1C	315.1C								
CHU57	R0a+60.1T	16126C	16362C	16519C	58C	60.1T	64T	263G	309.1C	315.1C								
CHU58	K1a1b1a	16223T	16224C	16234T	16311C	16519C	73G	114T	263G	309.1C	315.1C	497T						
CHU59	L3e2b+152	16172C	16183C	16189C	16320T	16519C	73G	150T	152C	195C	263G	315.1C						
CHU60	H1e1a6	16147T	16264T	16519C	150T	263G	315.1C											
CHU61	T	16126C	16294T	16304C	16519C	73G	263G	309.1C	315.1C									
CHU62	K1a	16129A	16224C	16256T	16311C	16519C	73G	263G	315.1C	497T								
CHU63	U1a1a1	16182C	16183C	16189C	16193.1C	16249C	73G	263G	285T	309.1C	309.2C	315.1C	385G	d523	d524	573.1C	573.2C	
CHU64	D1j	16223T	16242T	16311C	16325C	16362C	73G	152C	235G	263G	309.1C	315.1C	489C					
CHU65	U3	16343G	73G	150T	263G	315.1C												
CHU66	K1b1a1+199	16093C	16224C	16311C	16319A	16463G	16519C	73G	152C	199C	263G	309.1C	315.1C	524.1A	524.2C	524.3A	524.4C	
CHU67	U3a	16343G	16390A	16519C	73G	150T	263G	309.1C	309.2C	315.1C								
CHU68	H	16223T	16519C	263G	315.1C													
CHU69	R0a+60.1T	16126C	16362C	16519C	58C	60.1T	64T	263G	309.1C	315.1C								
CHU70	R0a+60.1T	16126C	16362C	16519C	58C	60.1T	64T	263G	309.1C	315.1C								
CHU71	R0a+60.1T	16126C	16362C	16519C	58C	60.1T	64T	263G	309.1C	315.1C								
CHU104	R0a+60.1T	16126C	16362C	16519C	58C	60.1T	64T	263G	309.1C	315.1C								
CHU105	L3e2b+152	16172C	16183C	16189C	16223T	16320T	16519C	73G	150T	152C	195C	263G	315.1C					
CHU106	U5b1d2	16239T	16270T	73G	150T	263G	315.1C											
CHU107	K1a	16129A	16224C	16311C	16519C	73G	263G	315.1C	497T									
CHU108	T2c1d	16126C	16292T	16294T	16519C	73G	146C	263G	279C	309.1C	315.1C							
CHU109	U1a1a1	16182C	16183C	16189C	16249C	73G	263G	285T	309.1C	309.2C	315.1C	385G	d523	d524	573.1C	573.2C		
CHU110	L3e2b+152	16172C	16183C	16189C	16223T	16320T	16519C	73G	150T	152C	195C	263G	315.1C					
CHU111	T2b23	16126C	16147T	16294T	16296T	16297C	16304C	16519C	73G	263G	309.1C	309.2C	315.1C					
CHU112	R0a+60.1T	16126C	16362C	16519C	58C	60.1T	64T	263G	309.1C	315.1C								
CHU113	R0a+60.1T	16126C	16362C	16519C	58C	60.1T	64T	263G	309.1C	315.1C								
CHU115	R0a+60.1T	16126C	16362C	16519C	58C	60.1T	64T	263G	309.1C	315.1C								
CHU117	M1a1	16129A	16183C	16189C	16223T	16249C	16311C	16359C	16519C	73G	195C	263G	315.1C	489C				
CHU118	K1a4a1	16129A	16224C	16256T	16311C	16519C	73G	263G	315.1C	497T								
CHU120	U1a1a1	16182C	16183C	16189C	16249C	73G	263G	285T	309.1C	309.2C	315.1C	385G	d523	d524	573.1C	573.2C		
CHU126	H1j8	16129A	16240G	16519C	152C	185A	263G	309.1C	315.1C									
CHU127	R0a+60.1T	16126C	16362C	16519C	58C	60.1T	64T	263G	309.1C	315.1C								
CHU128	T2c1d	16126C	16292T	16294T	16519C	73G	146C	263G	279C	309.1C	315.1C							
CHU129	T1a	16126C	16163G	16186T	16189C	16294T	16298C	16301T	16319A	16519C	73G	263G	309.1C	315.1C				
CHU130	T1a	16126C	16163G	16186T	16189C	16294T	16298C	16319A	16519C	73G	263G	309.1C	315.1C					
CHU131	HV0+195	16298C	72C	195C	263G	309.1C	309.2C	315.1C										
CHU132	R0a+60.1T	16126C	16362C	16519C	58C	60.1T	64T	263G	309.1C	315.1C								
CHU133	K1a1b1a	16223T	16224C	16234T	16311C	16519C	73G	114T	263G	315.1C	497T							

CHU134	U5b1f1a	16192T	16270T	16319A	73G	150T	263G	315.1C	533G									
CHU135	T1a	16126C	16163G	16186T	16189C	16294T	16298C	16319A	16519C	73G	263G	309.1C	315.1C					
CHU136	H66a	16172C	16519C	263G	315.1C													
CHU137	J2b1a	16069T	16126C	16193T	16278T	73G	150T	152C	263G	295T	315.1C	489C	523d	524d				
CHU139	T2c1d	16126C	16292T	16294T	16519C	73G	146C	263G	279C	309.1C	315.1C							
CHU140	T1a1'3	16126C	16163G	16186T	16189C	16294T	16519C	16527T	73G	152C	195C	263G	309.1C	315.1C	573.1C			
CHU141	K2b1a1a	16222T	16224C	16270T	16311C	16519C	73G	146C	195C	263G	315.1C							
CHU142	R0a+60.1T	16126C	16362C	16519C	58C	60.1T	64T	263G	309.1C	315.1C								
CHU143	T2b23	16126C	16147T	16294T	16296T	16297C	16304C	73G	263G	309.1C	309.2C	315.1C						
CHU144	U5a2	16192T	16256T	16270T	16526A	73G	263G	309.1C	315.1C									
CHU145	L3e2b+152	16172C	16183C	16189C	16223T	16320T	16335G	16519C	73G	150T	152C	195C	263G	315.1C				
CHU146	H6	16126C	16362C	16482G	16519C	239C	263G	309.1C	315.1C									
CHU147	T1a	16126C	16163G	16186T	16189C	16294T	16298C	16319A	16519C	73G	263G	309.1C	315.1C					
CHU148	T2b5a1	16126C	16294T	16304C	16519C	73G	152C	263G	309.1C	309.2C	315.1C	573.1C						
CHU149	H1n6	16519C	263G	309.1C	315.1C	552A												
CHU150	J2a1a1	16069T	16126C	16145A	16231C	16261T	73G	150T	152C	195C	203A	215G	263G	295T	315.1C	319C	489C	513A
CHU151	T2	16093C	16126C	16294T	16296T	16519C	73G	263G	315.1C									
CHU153	J1c2o	16069T	16126C	16163G	16266T	16311C	16519C	73G	185A	188G	204C	228A	263G	295T	315.1C	462T	489C	
CHU154	T	16126C	16294T	16304C	16519C	73G	263G	309.1C	309.2C	315.1C								
CHU155	K1a1b1a	16223T	16224C	16234T	16311C	16519C	73G	114T	263G	315.1C	497T							
CHU156	T1a	16126C	16163G	16186T	16189C	16294T	16298C	16319A	16519C	73G	263G	309.1C	315.1C					
CHU157	U1a1a1	16182C	16183C	16189C	16249C	16545C	73G	263G	285T	309.1C	309.2C	315.1C	385G	523d	524d	573.1C	573.2C	573.3C
CHU199	HV0+195	16298C	72C	195C	263G	309.1C	315.1C											
CHU200	L3e2b+152	16172C	16183C	16189C	16223T	16320T	16519C	73G	150T	152C	195C	263G	315.1C					
CHU201	R0a+60.1T	16126C	16362C	16519C	58C	60.1T	64T	263G	309.1C	315.1C								

*Parson, W. and A. Dür (2007). EMPOP--a forensic mtDNA database. Forensic Sci Int Genet 1(2): 88-92.

Supplementary Table 4. Mitochondrial D-loop haplotypes and haplogroups of the 79 Majorcan samples (EMPOP*: EMP00837), classified according to HaploGrep2 (Build 17, PhyloTree).

Sample	Haplogroup	Haplotypes																	
MAJ01	HV0	16298C	72C	263G	295T	315.1C													
MAJ02	H1+152	152C	263G	309.1C	315.1C	466C													
MAJ03	T2	16126C	16240G	16296T	16519C	73G	146C	263G	315.1C										
MAJ04	X2c	16093C	16169T	16183C	16189C	16223T	16255A	16278T	16519C	73G	153G	195C	225A	227G	263G	315.1C			
MAJ05	H13a1a2a	16278T	16519C	263G	309.1C	315.1C													
MAJ06	U5b2b1a1	16270T	16292T	16362C	16366T	73G	150T	263G	309.1C	315.1C									
MAJ07	H1bo	16519C	263G	267C	315.1C	485C													
MAJ08	K1a	16129A	16224C	16311C	16519C	73G	263G	315.1C	497T										
MAJ09	K1a4a1a+195	16093C	16224C	16311C	16519C	73G	195C	263G	315.1C	497T	d523	d524							
MAJ10	H1ag1a	16183C	16189C	16218T	16256T	16519C	263G	315.1C											
MAJ11	U5b2a2	16189C	16192T	16270T	16398A	73G	263G	315.1C											
MAJ13	U2e1e	16051G	16111T	16129C	16145A	16183C	16189C	16193.1C	16362C	16519C	73G	152C	217C	263G	309.1C	309.2C	315.1C	340T	508G
MAJ14	H1e5	16256T	16519C	263G	315.1C														
MAJ15	H11a	16257T	16293G	16311C	93G	195C	263G	315.1C											
MAJ17	K2b1a1	16224C	16270T	16311C	16519C	73G	146C	263G	315.1C										
MAJ18	H1bv1	16362C	16519C	263G	315.1C														
MAJ20	I2´3	16129A	16174T	16223T	16261T	16391A	16519C	73G	152C	199C	204C	207A	250C	263G	309.1C	309.2C	315.1C		
MAJ21	H3z	16189C	16294T	16362C	16519C	263G	293C	309.1C	315.1C										
MAJ22	H1+16189	16189C	16519C	57G	60.1T	263G	315.1C												
MAJ23	V+@16298	72C	263G	315.1C															
MAJ24	H1bv1	16104T	16362C	16519C	152C	263G	315.1C												
MAJ25	H1+152	16519C	152C	263G	309.1C	309.2C	315.1C												
MAJ26	H1+16189	16189C	16519C	263G	309.1C	315.1C													
MAJ27	HV0	16298C	72C	263G	309.1C	315.1C	d523	d524											
MAJ28	H1m1	16519C	146C	263G	315.1C														
MAJ29	U5b2b3	16224C	16270T	16519C	73G	150T	263G	309.1C	315.1C	517T									
MAJ30	H3w	16248T	16304C	146C	263G	309.1C	309.2C	315.1C											
MAJ31	J2b1a	16069T	16093C	16126C	16193T	16278T	73G	150T	152C	263G	295T	315.1C	489C						
MAJ32	I	16129A	16223T	16262T	16391A	16519C	73G	199C	204C	250C	263G	315.1C							
MAJ33	T2c1d	16126C	16292T	16294T	16519C	73G	146C	263G	279C	309.1C	315.1C								
MAJ34	K2b1a1	16224C	16270T	16311C	16519C	73G	146C	263G	315.1C										
MAJ35	K2b1a1	16224C	16270T	16311C	16519C	73G	146C	263G	315.1C										
MAJ37	HV0	16298C	64T	72C	263G	309.1C	315.1C	498.1C	d523	d524									
MAJ38	K1a	16224C	16311C	16519C	73G	263G	315.1C	497T	524.1A	524.2C	524.3A	524.4C	524.5A	524.6C					
MAJ39	HV0+195	16298C	72C	195C	263G	309.1C	315.1C	573.1C											
MAJ40	H1+152	16261G	16519C	152C	263G	309.1C	315.1C												
MAJ41	U2e2a2	16051G	16092C	16129C	16183C	16189C	16362C	16519C	16525G	73G	152C	217C	263G	315.1C	508G				
MAJ42	H57	16519C	64T	93G	146C	263G	315.1C												
MAJ43	U5b2b3	16224C	16270T	73G	150T	152C	263G	315.1C	517T										

MAJ44	J1b1b	16069T	16126C	16145A	16261T	16263C	16519C	73G	263G	271T	295T	309.1C	315.1C	462T	489C	d523	d524	
MAJ45	HV0	16298C	16311C	72C	73G	263G	309.1C	309.2C	315.1C									
MAJ46	H2a2a	16519C	263G	315.1C														
MAJ47	J1c	16069T	16126C	73G	228A	263G	295T	309.1C	315.1C	462T	489C							
MAJ48	HV15	16129A	16234T	16311C	16519C	263G	309.1C	315.1C										
MAJ49	U5b3	16270T	16304C	16399G	64T	73G	150T	204C	228A	263G	315.1C							
MAJ50	H24	16293G	309.1C	315.1C														
MAJ51	K1a	16224C	16304C	16311C	16519C	73G	263G	315.1C	497T									
MAJ52	K2a5	16224C	16311C	16519C	73G	146C	152C	263G	315.1C	324T								
MAJ53	T2a1b	16126C	16218T	16294T	16296T	16324C	16519C	73G	263G	315.1C								
MAJ54	N1b1	16145A	16176G	16187T	16223T	16311C	16390A	16519C	73G	152C	263G	315.1C						
MAJ55	L3d1b2	16124C	16223T	16519C	73G	150T	152C	263G	309.1C	315.1C	d523	d524						
MAJ56	T	16126C	16294T	16304C	16519C	73G	93G	199C	263G	315.1C								
MAJ57	L2a1b +143	16189C	16192T	16223T	16278T	16294T	16309G	16390A	73G	143A	146C	152C	195C	263G	309.1C	315.1C		
MAJ58	H5s	16111T	16304C	16311C	16391A	16519C	263G	309.1C	309.2C	315.1C	456T							
MAJ59	H3ak	16519C	143A	263G	309.1C	309.2C	315.1C											
MAJ60	U4b3	16278T	16356C	16519C	73G	195C	215G	263G	309.1C	315.1C	499A	524.1A	524.2C					
MAJ61	H11a2	16092C	16257T	16293G	16311C	93G	195C	263G	315.1C									
MAJ62	K2b1a1a	16222T	16224C	16270T	16311C	16519C	73G	146C	195C	263G	315.1C							
MAJ63	HV0	16298C	72C	263G	309.1C	315.1C												
MAJ64	K1b1 +(16093)	16093C	16192T	16224C	16311C	16319A	16519C	73G	152C	263G	309.1C	315.1C	524.1A	524.2C				
MAJ65	H1m1	16519C	146C	263G	315.1C													
MAJ66	H1e +16129	16129A	16311C	152C	263G	309.1C	315.1C	d523	d524									
MAJ67	HV0 +195	16298C	72C	195C	263G	309.1C	315.1C											
MAJ68	H24	16293G	309.1C	315.1C														
MAJ69	HV0	16298C	72C	263G	309.1C	315.1C												
MAJ70	H1j8	16129A	16240G	16519C	152C	185A	263G	309.1C	315.1C									
MAJ71	H6	16362C	16482G	16545C	239C	263G	309.1C	315.1C										
MAJ72	HV0 +195	16298C	72C	195C	228A	263G	309.1C	315.1C										
MAJ73	J1b1a1	16069T	16092C	16126C	16145A	16172C	16222T	16261T	73G	146C	242T	263G	295T	315.1C	462T	489C		
MAJ74	H1e +16129	16129A	16519C	263G	315.1C													
MAJ75	H1m1	16519C	146C	263G	315.1C													
MAJ76	U2e1'2'3	16051G	16129C	16183C	16189C	16274A	16362C	16519C	73G	152C	217C	263G	309.1C	309.2C	315.1C	508G	524.1A	524.2C
MAJ77	T2a1b	16092C	16126C	16294T	16296T	16324C	16519C	73G	263G	315.1C								
MAJ78	H2a5a1	16291T	16519C	217C	263G	315.1C												
MAJ79	H6	16362C	16482G	239C	263G	309.1C	315.1C											
MAJ80	HV4a2a	16287T	16519C	263G	309.1C	315.1C												
MAJ81	H6	16362C	16482G	239C	263G	315.1C												
MAJ104	H1e +16129	16129A	16311C	152C	263G	309.1C	315.1C	d523	d524									
MAJ198	U5b3	16192T	16270T	16304C	16526A	73G	150T	228A	263G	309.1C	315.1C							

*Parson, W. and A. Dür (2007). EMPOP--a forensic mtDNA database. Forensic Sci Int Genet 1(2): 88-92.

Supplementary Table 5. Populations and references used for the different analysis performed in this work.

Marker	Population	Reference
Y-SNPs	Chuetas	Present Study
	Sephardic 1	Behar et al. (2010) Nature 466: 238–242
	Sephardic 2	Adams et al. (2008) Am. J. Hum. Genet. 83: 725–736
	Bragança Jews	Nogueiro et al. (2010) Am. J. Phys. Anthropol. 141: 373–381
	Ashkenazi 1	Behar et al. (2010) Nature 466: 238–242
	Ashkenazi 2	Behar et al. (2004) Hum. Genet. 114: 354–365
	Azerbaijan Jews	Behar et al. (2010) Nature 466: 238–242
	Cochin Jews	Chaubey et al. (2016) Sci. Rep. 6: 19166
	Cohanim	Hammer et al. (2009) Hum. Genet. 126: 707–717
	Ethiopia Jews 1	Cruciani et al. (2002) Am. J. Hum. Genet. 70(5): 1197–1214
	Ethiopia Jews 2	Behar et al. (2010) Nature 466: 238–242
	Georgia Jews	Behar et al. (2010) Nature 466: 238–242
	Iran Jews	Behar et al. (2010) Nature 466: 238–242
	Iraq Jews	Behar et al. (2010) Nature 466: 238–242
	Israelian	Hammer et al. (2009) Hum. Genet. 126: 707–717
	Libya Jews	Shen et al. (2004) Hum. Mutat. 24:248–260
	Morocco Jews	Behar et al. (2010) Nature 466: 238–242
	Mumbai Jews	Behar et al. (2010) Nature 466: 238–242
	Uzbekistan Jews	Behar et al. (2010) Nature 466: 238–242
	Majorca 1	Present Study
	Majorca 2	Adams et al. (2008) Am. J. Hum. Genet. 83: 725–736
	Andalusia	Adams et al. (2008) Am. J. Hum. Genet. 83: 725–736
	Basque Country	Adams et al. (2008) Am. J. Hum. Genet. 83: 725–736
	Castile	Adams et al. (2008) Am. J. Hum. Genet. 83: 725–736
	Catalonia	Adams et al. (2008) Am. J. Hum. Genet. 83: 725–736
	Extremadura	Adams et al. (2008) Am. J. Hum. Genet. 83: 725–736
	Galicia	Adams et al. (2008) Am. J. Hum. Genet. 83: 725–736
	Valencia	Adams et al. (2008) Am. J. Hum. Genet. 83: 725–736
	Portugal North	Adams et al. (2008) Am. J. Hum. Genet. 83: 725–736
	Tras-os-montes	Nogueiro et al. (2010) Am. J. Phys. Anthropol. 141: 373–381
	Georgia	Bataglia et al. (2009) Eur. J. Hum. Genet. 17: 820–830
	Hungary	Bataglia et al. (2009) Eur. J. Hum. Genet. 17: 820–830
	Moldova	Varzaki et al. (2013) PLoS One, 8(1): e53731
	Poland	Bataglia et al. (2009) Eur. J. Hum. Genet. 17: 820–830
	Ukraine	Varzaki et al. (2013) PLoS One, 8(1): e53731
	Iran	Crugni et al. (2012) PLoS One, 7(7): e41252
	Iraq	Al-Zahery et al. (2011) BMC Evol. Biol. 11(1): 1–16
	Jordan (Ammam)	Flores et al. (2005) J. Hum. Genet. 50(9): 435–441
	Jordan (Death Sea)	Flores et al. (2005) J. Hum. Genet. 50(9): 435–441
	Palestinian 1	Flores et al. (2005) J. Hum. Genet. 50(9): 435–441
	Palestinian 2	Behar et al. (2010) Nature 466: 238–242
	Yemen	Cadenas et al. (2008) Eur. J. Hum. Genet. 16: 374–386
	Uzbekistan	Behar et al. (2010) Nature 466: 238–242
	India (South)	Behar et al. (2010) Nature 466: 238–242
	Algeria	Bekada et al. (2015) PLoS One, 10(9): e0138453
	Egypt	Luis et al. (2004) Am. J. Hum. Genet. 74: 532–544
	Morocco	Bosch et al. (2001) Am. J. Hum. Genet. 68(4). 1019–1029
	Morocco (Arabs)	Cruciani et al. (2002) Am. J. Hum. Genet. 70(5): 1197–1214
	Morocco (Berbers)	Cruciani et al. (2002) Am. J. Hum. Genet. 70(5): 1197–1214
	Sahrawi	Bosch et al. (2001) Am. J. Hum. Genet. 68(4). 1019–1029
Y-STR	Chuetas	Present Study
	Bragança Jews	Nogueiro et al. (2010) Am. J. Phys. Anthropol. 141: 373–381
	Majorca	Present Study
	Barcelona	Sánchez et al. (2007) Forensic Sci. Int. 172: 211–217
	Bosnia-Herzegovina	Kovacekic et al. (2013) Croat. Med. J. 54: 286–290
	Bulgaria	Karachanak et al. (2013) PLoS One, 8(3): e56779
	East Tyrol	Niedersttater et al. (2012) PLoS One, 7(7): e41885
	Greece	Kovatsi et al. (2013) Forensic Sci. Int. Genet. 4: e21–e22
	Italy	Onofri et al. (2007) Int. J. Legal Med. 121: 234–237
	Montenegro	Mirabal et al. 2010) Am. J. Phys. Anthropol. 142: 380–390
	Romania	Stanciu et al. (2010) Leg. Med. 12: 259–264
	Russia	Roewer et al. (2008) Int. J. Legal Med. 122: 219–223
	Serbia	Vaselinovik et al. (2008) Forensic Sci. Int. 176: e23–e28
	Ukraine	Mielnik-Sikorska et al. (2013) Forensic Sci. Int. Genet. 7: 200–203

	USA caucasic	Coble et al. (2013) Forensic Sci. Int. Genet. 7: e66–e68
	USA hispanic	Coble et al. (2013) Forensic Sci. Int. Genet. 7: e66–e68
	Anatolia	Alakoc (2010) Forensic Sci. Int. Genet. 4: e135–e137
	Armenia	Lowery et al. (2013) Legal Med. 15: 85–90
	Iran	Roewer et al. (2009) Forensic Sci. Int. Genet. 4: e53–e55
	Lebanon	Haber et al. (2011) J. Hum. Genet. 56(1): 29–33
	Pathans	Lee et al. (2014) Forensic Sci. Int. Genet. 11:111–116
	Libya	Fadhlaoui-Zid et al. (2013) PLoS One 8(11): e80293
	East Libya	Elmrghni et al. (2012) Forensic Sci. Int. Genet. 6(2): 224–227
	West Libya	Triki-Fendry et al. (2013) Forensic Sci. Int. Genet. 7: e59–e61
	Morocco	Fadhlaoui-Zid et al. (2013) PLoS One 8(11): e80293
mtDNA	Chuetas	Present Study
	Sephardic Jews	Behar et al (2008) PLoS One 3(4): e2062
	Bragança Jews	Nogueiro et al. (2015) Eur. J. Hum. Genet. 23(5): 693–699
	Ashkenazi 1	Behar et al. (2006) Am. J. Hum. Genet. 78(3): 487–497
	Ashkenazi 2	Picornell et al. (2006) Int. J. Legal Med. 120(5): 271–281
	Iran Jews	Behar et al (2008) PLoS One 3(4): e2062
	Yemen Jews 1	Thomas et al. (2002) Am. J. Hum. Genet. 70(6): 1411–1420
	Yemen Jews 2	Behar et al (2008) PLoS One 3(4): e2062
	Yemen Jews 3	Černý et al. (2011) Mol. Biol. Evol. 28(1): 71–78
	Ethiopia Jews 1	Thomas et al. (2002) Am. J. Hum. Genet. 70(6): 1411–1420
	Ethiopia Jews 2	Behar et al (2008) PLoS One 3(4): e2062
	North Africa Jews 1	Picornell et al. (2006) Int. J. Legal Med. 120(5): 271–281
	North Africa Jews 1	Behar et al (2008) PLoS One 3(4): e2062
	Majorca	Present Study
	Valencia	Santos et al. (2014) Am. J. Hum. Biol. 26(2):130–141
	Galicia	Santos et al. (2014) Am. J. Hum. Biol. 26(2):130–141
	Portugal 1	Santos et al. (2014) Am. J. Hum. Biol. 26(2):130–141
	Portugal 2	Marques et al. (2015) Forensic Sci. Int. Genet. 15:27–32
	Italy	Messina et al. (2015) Am. J. Hum. Boil. 27(4): 508–519
	Italy (Tuscany)	Achilli et al (2007) Am. J. Hum. Genet. 80(4): 759–768
	Bulgaria	Karachanak et al. (2012) Int. J. Legal Med. 126 (4): 497–503
	France	Badro et al. (2013) PLoS One 8(1): e54616
	Greece	Badro et al. (2013) PLoS One 8(1): e54616
	Georgia 1	Thomas et al. (2002) Am. J. Hum. Genet. 70(6): 1411–1420
	Georgia 2	Quintana-Murci et al. (2004) Am. J. Hum. Genet. 74(5): 827–845
	Balkans	González et al. (2008) Ann. Hum. Biol. 35(2) 212–231
	Turkey 1	Quintana-Murci et al. (2004) Am. J. Hum. Genet. 74(5): 827–845
	Turkey 2	González et al. (2008) Ann. Hum. Biol. 35(2) 212–231
	Jordania (Ammam)	González et al. (2008) Ann. Hum. Biol. 35(2) 212–231
	Jordania	Badro et al. (2013) PLoS One 8(1): e54616
	Bedouins 1	Behar et al (2008) PLoS One 3(4): e2062
	Bedouins 2	Černý et al. (2011) Mol. Biol. Evol. 28(1): 71–78
	Druze 1	Behar et al (2008) PLoS One 3(4): e2062
	Druze 2	González et al. (2008) Ann. Hum. Biol. 35(2) 212–231
	Lebanon	Badro et al. (2013) PLoS One 8(1): e54616
	Iran	Derenko et al. (2013) PLoS One 8(11): e80673
	Iran (Zagros Mountains)	Quintana-Murci et al. (2004) Am. J. Hum. Genet. 74(5): 827–845
	Iraq 1	Al-Zahery et al. (2011) BMC Evol. Biol. 11(1): 1–16
	Iraq 2	Badro et al. (2013) PLoS One 8(1): e54616
	Palestine 1	Behar et al (2008) PLoS One 3(4): e2062
	Palestine 2	González et al. (2008) Ann. Hum. Biol. 35(2) 212–231
	Palestine 3	Badro et al. (2013) PLoS One 8(1): e54616
	Syria 1	Thomas et al. (2002) Am. J. Hum. Genet. 70(6): 1411–1420
	Syria 2	Badro et al. (2013) PLoS One 8(1): e54616
	Pakistan	Quintana-Murci et al. (2004) Am. J. Hum. Genet. 74(5): 827–845
	Turkmenistan	González et al. (2008) Ann. Hum. Biol. 35(2) 212–231
	Kuwait	Sheible et al. (2011) Forensic Sci. Int. Genet. 5(4): e112–e113
	Arabia Saudi 1	Černý et al. (2011) Mol. Biol. Evol. 28(1): 71–78
	Arabia Saudi 1	Badro et al. (2013) PLoS One 8(1): e54616
	Yemen 1	Thomas et al. (2002) Am. J. Hum. Genet. 70(6): 1411–1420
	Yemen 2	Černý et al. (2011) Mol. Biol. Evol. 28(1): 71–78
	Yemen 3	Badro et al. (2013) PLoS One 8(1): e54616
	Ethiopia 1	Thomas et al. (2002) Am. J. Hum. Genet. 70(6): 1411–1420
	Ethiopia 2	Černý et al. (2011) Mol. Biol. Evol. 28(1): 71–78
	Ethiopia 3	Badro et al. (2013) PLoS One 8(1): e54616
	Sudan	Černý et al. (2011) Mol. Biol. Evol. 28(1): 71–78

Sudan (Nubia)	González et al. (2008) Ann. Hum. Biol. 35(2) 212–231
Chad	Černý et al. (2011) Mol. Biol. Evol. 28(1): 71–78
Egypt 1	Badro et al. (2013) PLoS One 8(1): e54616
Egypt 2	Elmadawy et al. (2013) Legal Med. 15(6): 338–341
Libya	Badro et al. (2013) PLoS One 8(1): e54616
Tunisia	Černý et al. (2011) Mol. Biol. Evol. 28(1): 71–78
Morocco 1	Černý et al. (2011) Mol. Biol. Evol. 28(1): 71–78
Morocco 2	Aboukhalid et al. (2013) Int. J. Legal Med. 127(4): 757–759
Morocco 3	Badro et al. (2013) PLoS One 8(1): e54616

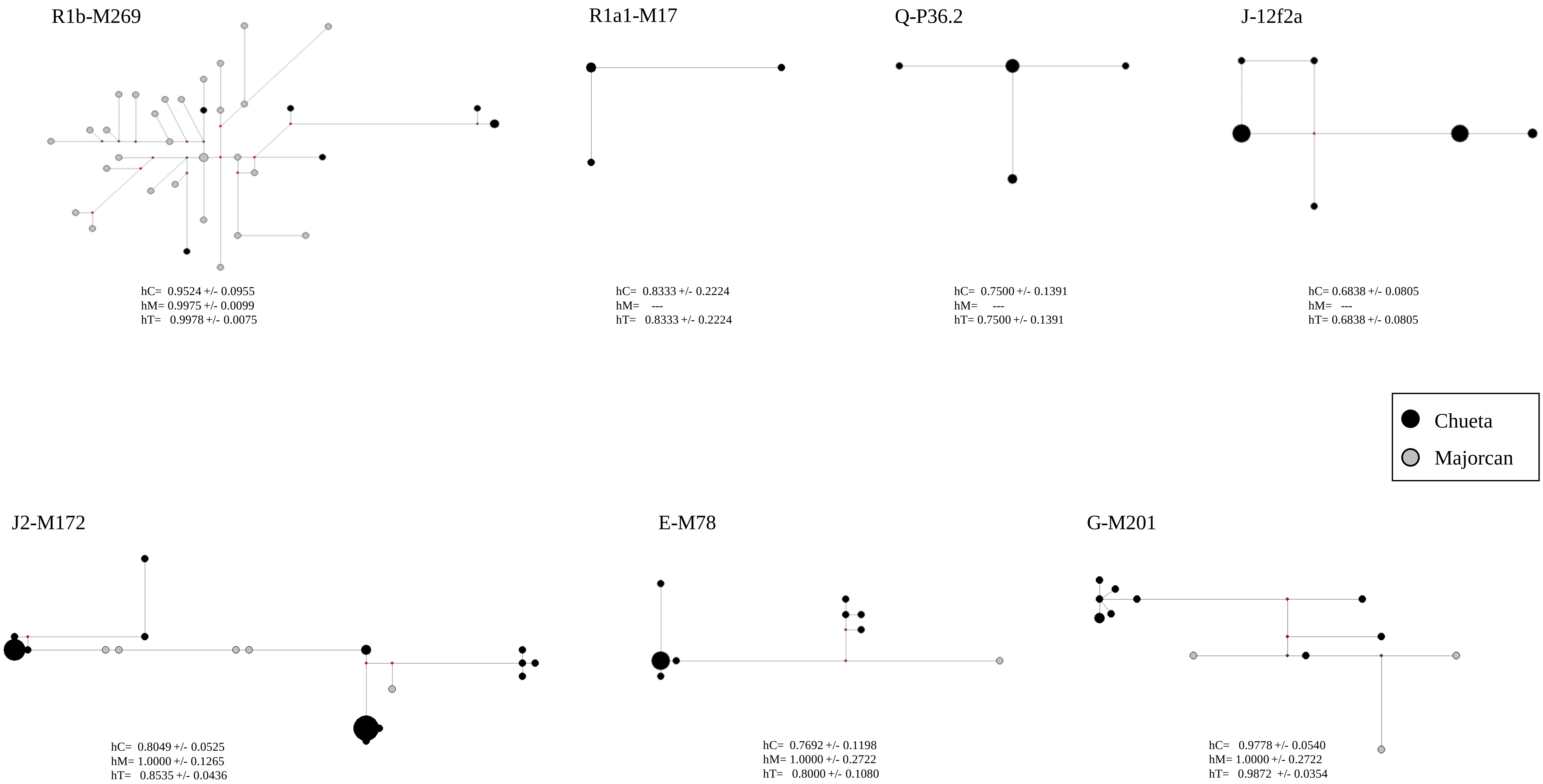

Supplementary Figure 1. Networks based on Y-STR haplotypes of the main haplogroups found in Chueta (black) and Majorcan (grey) populations, as one of their parental populations. Haplotype diversities (HD) in each haplogroup are shown as follows; hC: HD in Chuetas, hM: HD in Majorcans, and finally hT: total HD within each haplogroup. No putative parental Jewish population was used in these analyses due to the lack of data for this set of markers in any proper population.
